# Research and application of pre-excavated double withdrawal channel technology without coal pillar abandonment

**DOI:** 10.1038/s41598-026-47412-x

**Published:** 2026-04-10

**Authors:** Jiao Zhang, Zhan Shi, Chao Wu, FuKun Xiao, Lei Shi

**Affiliations:** 1 Present Address: Heilongjiang University of Science and Technology, Harbin, 150022 China; 2 Heilongjiang Ground Pressure & Gas Control in Deep Mining Key Lab, Heilongjiang University of Science and Technology, Harbin, 150022 China; 3 Jinneng Holding Coal Industry Group Co., Ltd. Datong Coal Mine Group Madaotou Coal Industry Co., Ltd., Datong, 037003 China; 4 Lvliang Engn Res Ctr Intelligent Coal Mine, Lvliang, 033001 China

**Keywords:** Withdrawal channel, Recovery of coal pillar, Two functions of a roadway, Stability of surrounding rock, Deviatoric stress, Energy science and technology, Engineering, Solid Earth sciences

## Abstract

In order to solve the problem that the surrounding rock control of the pre-excavation double-retracement channel is difficult and a large number of coal pillars cannot be recovered. Taking chuancaogeban coal mine as the engineering background, the mechanical model in the process of gradual connection between coal mining face and main withdrawal channel is constructed, and the response characteristics of surrounding rock in withdrawal channel are simulated and verified by FLAC3D numerical calculation model. The instability mechanism of this kind of roadway is revealed, and a comprehensive stability control technology of surrounding rock and the technology of recovering all coal pillars are proposed, and the field engineering verification is carried out. The results show that when the coal mining face is close to the main retracement channel, when the distance between the coal mining face and the main retracement channel is 4 m, the coal body between the coal mining face and the main retracement channel enters an unstable state. Using 8 m wide filling wall to replace the coal pillar between the main retracement channel and the auxiliary retracement channel can ensure the stability of the surrounding rock of the two retracement channels after the coal mining face passes through the main retracement channel. In addition, the retained auxiliary withdrawal channel also has the functions of auxiliary production and coal pillar recovery, and finally realizes the technology of pre-excavation double withdrawal channel without coal pillar abandonment. As the coal mining face gradually approaches the main retracement channel, the surrounding rock stress of the main retracement channel and the auxiliary retracement channel has significant dynamic development. Based on the development law of the deviatoric stress of the surrounding rock, the combined control technology of ‘ pre-cutting roof + high water material pillar + grouting reinforcement + single pillar + metal mesh + steel ladder belt + bolt + anchor cable ‘ in different regions is proposed. The field monitoring results show that during the removal of coal mining equipment, the surrounding rock deformation of the withdrawal channel is small, the surrounding rock control effect is good, and the equipment is safely and efficiently removed; during the recovery of coal pillars, the surrounding rock deformation of the retained auxiliary withdrawal channel meets the needs of production. The recoverable resources of coal seams will increase by 747,000t, and the economic benefits will increase by 117 million yuan.

## Introduction

After the working face is stopped, the production equipment can be quickly withdrawn to alleviate the continuous tension of the working face. More and more large coal mines adopt the layout of pre-excavation double withdrawal channels. The working face gradually approaches the main withdrawal channel until the main withdrawal channel is connected, so the withdrawal channel can be regarded as a special roadway. Before the main retracement channel is connected, both the main retracement channel and the auxiliary retracement channel are affected by the moving abutment pressure^1^. After the main retracement channel is penetrated, the auxiliary retracement channel is affected by the fixed bearing pressure^2^. Aiming at the problem that the surrounding rock of the retracement roadway is difficult to control, domestic and foreign scholars have carried out a lot of research.

In the study of the dynamic pressure of surrounding rock before the main retracement channel is penetrated, many scholars focus on the influence of roof fracture, pressure characteristics and mining stress on the stability of surrounding rock. Its essence is to control the influence of mining stress on the retracement channel. By optimizing the mining process parameters, the control of the deformation of the surrounding rock of the retracement channel is realized. For example, Wang^3^ adopted the technology of stopping mining and yielding pressure, adjusted the position and time of stopping mining in the working face, changed the position of periodic pressure, and reduces the length of periodic pressure. By artificially intervening the roof fracture position, He^4^ made the roof fracture position more conducive to the stability of surrounding rock. Yang^5^ reduced the continuous length of periodic pressure by reducing the advancing speed of coal mining face, and avoided the threat of roof pressure when the main retracement channel was connected. Lv^6^ reduced the disturbance of dynamic pressure of main roof by controlling mining height. Li^7^ adopted the slope adjustment of the working face to connect the main retracement channel, which avoided the large area connection between the working face and the main retracement channel.

On the study of the fixed abutment pressure of the surrounding rock of the auxiliary withdrawal channel after the main withdrawal channel is connected, many scholars focus on the stability of the protective coal pillar between the main and auxiliary withdrawal channels. By optimizing the size of the protective coal pillar, the deformation control of the surrounding rock of the auxiliary withdrawal channel is realized^8,9^. For example, Yang^10^ used the method of engineering measurement to analyze the fracture position of the roof and the critical instability width of the coal pillar, determined the position of the withdrawal channel, and obtained the reasonable width of the protective coal pillar of the withdrawal channel. Gao^11^ used the limit equilibrium theory to quantitatively calculate the width of the plastic zone under the influence of the main withdrawal channel and the auxiliary withdrawal channel, and determined the reasonable size of the protective coal pillar of the withdrawal channel. Liu^12^ analyzed the dynamic change of the working face, and believed that the dynamic change process of the coal pillar was mainly divided into three stages, namely, the stable stage, the stress increase stage and the stress transfer stage, and finally determined the size of the coal pillar between the withdrawal channels. Dang^13^ used GRT-101 polymer to reinforce the protective coal pillar of the withdrawal channel, which not only controlled the deformation of the surrounding rock of the withdrawal channel, but also reduced the size of the protective coal pillar. Zhang^14^ used concrete material to replace part of the coal pillar, which ensured the stability of the protective coal pillar of the withdrawal channel.

On the other hand, on the basis of optimizing the stability of the protective coal pillar, many scholars have also focused on the surrounding rock support technology of the auxiliary retracement channel. By optimizing the support parameters, the control of the surrounding rock deformation of the retracement channel has been realized, and the support structure has also changed from a single support structure to different combined support structures in different regions^15,16^. For example, Song^17^ studied the overburden structure and roof load characteristics of the working face, and determined the combined support scheme forming a safe and efficient surrounding rock support technology system for the retracement channel. Huang^18^ aimed at the support problem of the withdrawal channel under the action of high concentrated stress, and analyzed the deformation characteristics of the surrounding rock of the high-stress hard roof withdrawal channel, and proposed a high-strength partition surrounding rock control technology. Zhao^19^ proposed a secondary strengthening support based on the influence of the working advance support stress on the surrounding rock of the retracement channel. Li^20^ summarized the mine pressure behavior law, stress distribution law and roof deformation and failure characteristics in the advancing stage of the working face. Based on this, the surrounding rock grouting reinforcement, roof reinforcement, and the surrounding rock control technology of the retracement channel to improve the anchoring force of the anchor rod were proposed.

Domestic and foreign scholars have carried out a lot of research on the surrounding rock control of the double withdrawal channel, but there are still a large number of coal pillars around the double withdrawal channel that cannot be recovered, resulting in a large amount of coal resources. Aiming at this problem, this paper takes chuancaogeban coal mine as the engineering background, and proposes a technology that the coal pillar resources around the double withdrawal channel can be fully recovered. Based on the development law of the deviatoric stress of the surrounding rock of the double retracement channel, the joint control technology of ‘ pre-cut roof + high water material pillar + grouting reinforcement + single pillar + metal mesh + steel ladder belt + anchor rod + anchor cable ‘ in different regions is designed. And the above research results are applied to field practice.

## Project overview

**Fig. 1:**
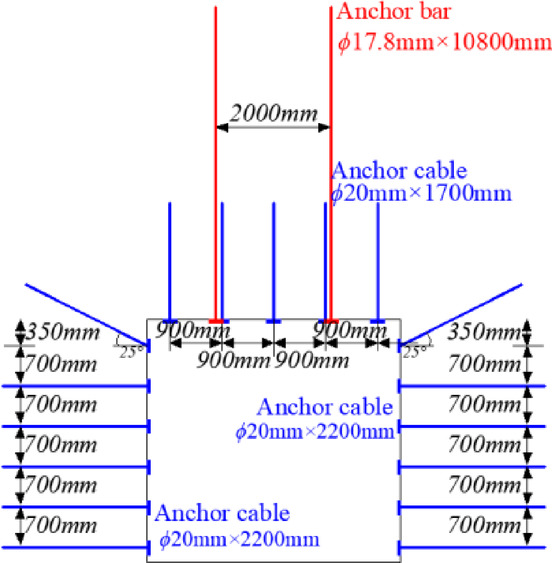
Surrounding rock support structure of main and auxiliary withdrawal channels in 3301 working face.

The average thickness of 3# coal seam in chuancaogeban Coal Mine is 4.2 m, and the buried depth is between 291 and 315 m, with an average buried depth of 312 m. In the 3# coal seam, three main roadways are established along the coal seam’s floor, with eight coal mining working faces, namely the 3301 through 3308 working faces. The average width of the 3301 to 3308 working faces is 198 m. The cross-sectional dimensions of the main roadway, the main withdrawal channel, and the auxiliary withdrawal channel are 4.4 m in width and 4.2 m in height. The size of the coal pillar between the main roadways and the size of the stop-mining protective coal pillar are both 44 m. The size of interval coal pillar between the main and auxiliary withdrawal channels is 28 m, while the size of coal pillar between the working faces is 30.8 m. The 3301 coal mining working face has been fully mined out. During the end mining stage of the 3301 coal mining working face, the deformation of the main and auxiliary withdrawal channels reachs 452 mm to 875 mm, with local spalling observed in some areas. The surrounding rock support structure of the main and auxiliary withdrawal channels in the 3301 coal mining working face is shown in Fig. 1.

**Fig. 2:**
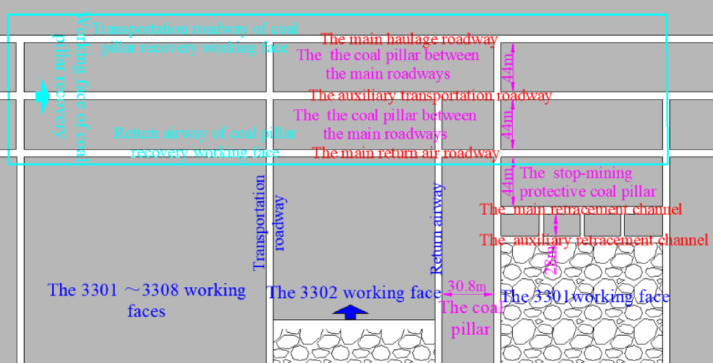
Layout diagram of the Working Face of 3# coal seam.

**Fig. 3:**
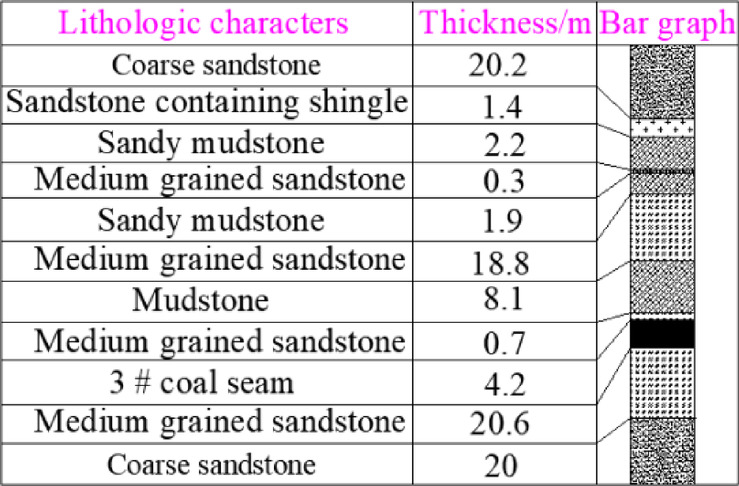
The lithology of roof and floor of 3 # coal seam.

The 3301 ~ 3308 working face follows a sequential mining approach. Once the mining of the 3301 ~ 3308 working faces is completed, the coal pillar recovery working face will be set up to extract the remaining coal pillars. The original main haulage roadway and the main return air roadway will function as the haulage roadway and return airway for the coal pillar recovery face, respectively. The layout of roadways and working faces in the 3# coal seam is shown in Fig. 2, and the lithology of the roof and floor strata of the 3# coal seam is illustrated in Fig. 3.

In summary, during the end mining phase of the 3# coal seam’s working face, significant deformation occurs in the surrounding rock of the withdrawal channel. Furthermore, only a portion of the remaining coal pillars is extracted, leaving a substantial number of coal resources.

## Size setting of interval coal pillar

**Fig. 4:**
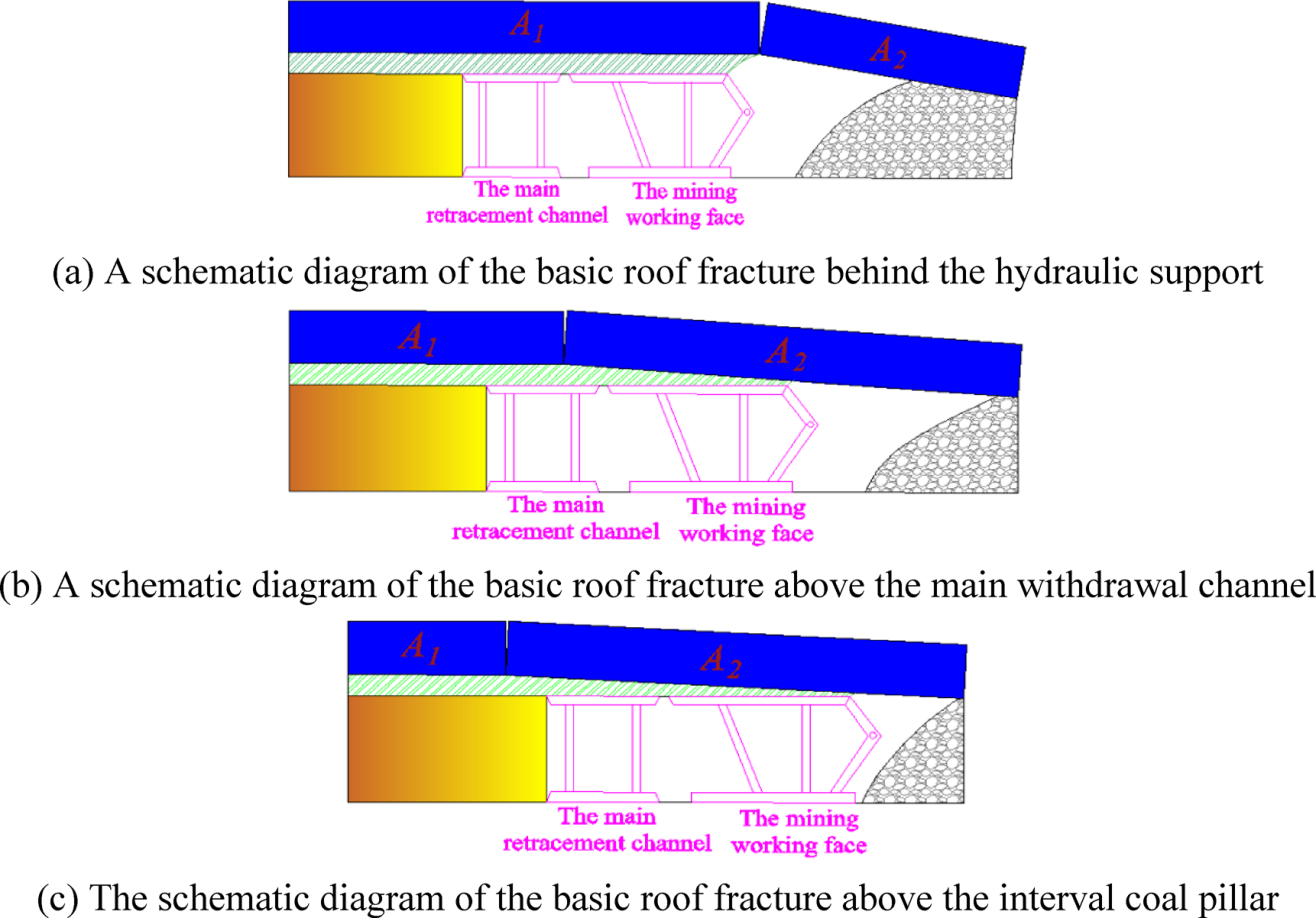
The basic roof fracture form.

In order to ensure the smooth operation of the migration equipment work in the end mining stage of the coal mining working face, technical measures are adopted to adjust the fracture position of roof. After the connection between the coal mining working face and the main withdrawal channel, according to the different fracture positions of the overlying basic roof, the fracture structure of the overlying basic roof of the main withdrawal channel can be categorized into three types^21–23^, as shown in Fig. 4.

Figure 4a shows the spatial structure of the rock block *A*_*2*_ in the basic roof, which is broken behind the hydraulic support. The rock mass *A*_*1*_ is not broken, and the deformation of the main withdrawal channel is determined by the rock mass *A*_*1*_. Figure 4b shows the spatial structure of the rock block *A*_*2*_ in the basic roof broken above the main withdrawal channel area. The structure is that the movement trend of the rock mass *A*_*1*_ and the broken rock block *A*_*2*_ jointly affect the deformation of the main withdrawal channel, and the surrounding rock of the main withdrawal channel is difficult to control. Figure 4c shows the spatial structure of the rock block *A*_*2*_ in the basic roof broken above the interval coal pillar (the coal pillar between the main withdrawal channel and the auxiliary withdrawal channel). The movement trend of the rock block *A*_*2*_ determines the deformation of the main withdrawal channel, and the surrounding rock control of the main withdrawal channel is difficult. The analysis above indicates that the spatial fracture structure in Fig. 4a is good for the stability of the surrounding rock of the main withdrawal channel.

**Fig. 5:**
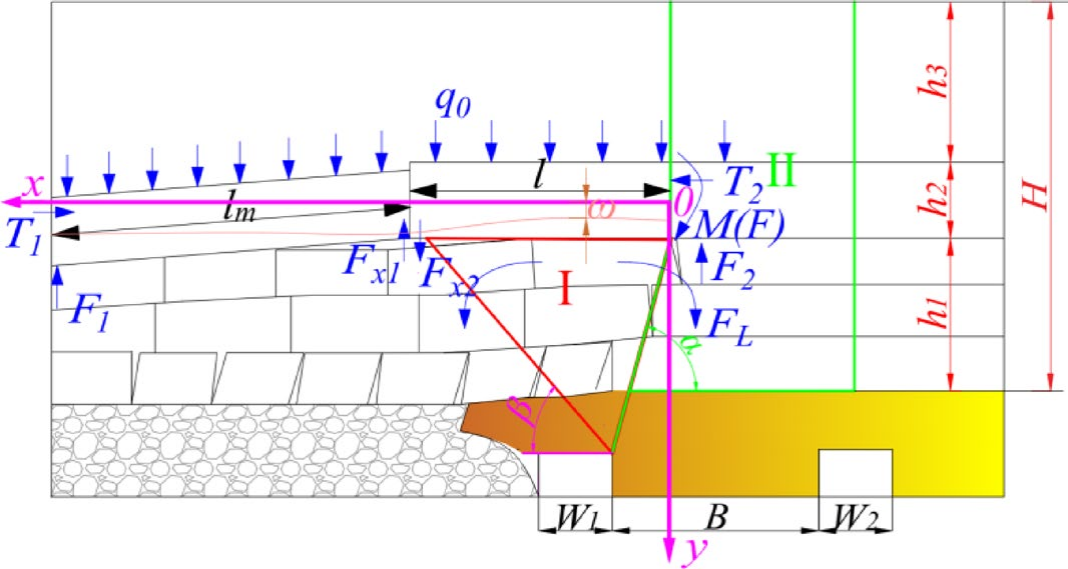
The spatial structure of the stop-mining position.

In the process of the coal mining working face gradually approaching the position of the main withdrawal channel, some technical means can be used to adjust the position of the basic roof fracture, so that the fracture position of the basic roof is most conducive to the stability of the surrounding rock of the main withdrawal channel. Therefore, the fracture structure of the overlying strata of the main withdrawal channel can be regarded as a masonry beam structure. When the coal mining working face is connected to the main withdrawal channel, the spatial structure around the main withdrawal channel, the auxiliary withdrawal channel and the interval coal pillar is shown in Fig. 5.

As shown in Fig. 5, the width of the interval coal pillar between the main withdrawal channel and the auxiliary withdrawal channel is *B*. The width of the main withdrawal channel is *W*_*1*_. The width of the auxiliary withdrawal channel is *W*_*2*_. The thickness of the direct roof is *h*_*1*_. The thickness of basic roof and strata controlled by basic roof is *h*_*2*_. The distance between the upper boundary of the basic roof control rock layer and the surface is *h*_*3*_. The comprehensive movement angle of the overlying rock and the contact angle of the broken rock layer in the goaf are *α* and *β*, respectively. The length of the basic roof overhang is *l*. The length of the fractured rock mass of the basic roof is *l*_*m*_, and the load of the overlying rock layer on the thick and hard rock layer is *q*_*0*_. At the stopping position of the working face, the interval coal pillar and the the cantilever rock beam form a "┤"-shaped spatial structure. In this "┤"-shaped spatial structure, the interval coal pillar serves as the vertical support, while the articulated structure of the cantilever rock beam and fractured rock blocks acts as the primary horizontal load-bearing body for transferring the static pressure. The hinged structure between the cantilever rock beam and the broken rock block is squeezed by the rock mass on both sides, forming horizontal thrust* T*_*1*_ and *T*_*2*_. In the vertical direction, the position of the broken rock block contacting the waste rock and the position of the supporting force provided to the cantilever rock beam are simplified as the concentrated force *F*_*1*_, *F*_*2*_, respectively. The deflection of the cantilever rock beam structure is *ω*. Assuming that the slow deformation of the "┤"-shaped spatial type structure can be approximated as a balanced structure, the mechanical equilibrium condition is established :

1 $$\left\{ \begin{gathered} \sum {F\left( x \right) = 0} \hfill \\ \sum {F\left( y \right) = 0} \hfill \\ \sum {M\left( F \right) = 0} \hfill \\ \end{gathered} \right.$$

The adjacent rock beams are squeezed each other, forming a pair of horizontal thrusts in the horizontal direction and satisfying the following equilibrium conditions :

2 $$T_{1} = T_{2}$$

Ignoring the influence of the rotation of the broken rock mass, the expression of the equilibrium of the hinged structure composed of the concentrated force *F*_*2*_ of the fixed support point, the concentrated force *F*_*1*_ of the contact gangue point, the cantilever rock beam and the broken rock mass is as follows :

3 $$F_{1} + F_{2} = \left( {q_{0} + \gamma_{2} h_{2} } \right)\left( {l + l_{m} } \right)$$

where *γ*_*2*_ is the bulk density of basic roof and strata controlled by basic roof.

The mutual extrusion of adjacent rock beams forms a pair of friction *F*_*X1*_ and friction *F*_*X2*_ in the vertical direction. The expression of the mechanical balance of the broken rock in the vertical direction is as follows :

4 $$\left\{ \begin{gathered} F_{1} + F_{x1} = \left( {q_{0} + \gamma_{2} h_{2} } \right)l_{m} \hfill \\ F_{x1} = F_{x2} \hfill \\ \end{gathered} \right.$$

By combining expressions (1) ~ (4), the expression of the concentrated force *F*_*2*_ is obtained as follows :

5 $$F_{2} = \frac{1}{2}\left( {q_{0} + \gamma_{2} h_{2} } \right)\left( {l + l_{m} } \right)$$

According to the area of area *II* in Fig. 5, the rock gravity *G* is estimated as follows :

6 $$G = \frac{{\gamma h_{1} }}{2}\left( {2B + W_{2} - h_{1} \cot \alpha } \right) + \gamma \left( {h_{2} + h_{3} } \right)\left( {B + \frac{{W_{2} }}{2} - h_{1} \cot \alpha } \right)$$

where *γ* is the average bulk density of rock strata.

The area *I* in Fig. 5 is the collapsed rock block under the hard rock stratum. Due to the existence of the hard rock stratum, this part of the rock block can still form a hinged structure at the stop mining boundary, and the weight of the area *I* is transmitted to the coal pillar and the collapsed gangue respectively. *F*_*L*_ is approximately half of the weight of region I, and its expression is as follows :

7 $$F_{L} = \frac{{\gamma_{1} h_{1} }}{4}\left( {h_{1} \cot \alpha + h_{1} \cot \beta } \right)$$

where *γ*_*1*_ is the bulk density of immediate roof.

The total weight *W* borne by the interval coal pillar is expressed as:

8 $$W = G + F_{2} + F_{L}$$

Under static conditions, the expression of the average stress *P* borne by the interval coal pillar is as follows:

9 $$P = \frac{W}{B} = \gamma H + \frac{\gamma H}{B}\left( {\frac{{W_{2} }}{2} - h_{1} \cot \alpha } \right) + \frac{{\left( {q_{0} + \gamma_{2} h_{2} } \right)\left( {l + l_{m} } \right)}}{2B} + \frac{{\gamma_{1} h_{1}^{2} \left( {\cot \beta + 3\cot \alpha } \right)}}{4B}$$

Both engineering practice and theoretical research show that the movement of the basic roof can produce dynamic load effect during the mining process of the coal working mining face. The basic roof not only produces flexural deformation but also accumulates energy. The relationship between the accumulated elastic energy *U* and the deflection *ω* of the rock beam with the basic roof overhanging length *l* is expressed as follows:

10 $$U = \int_{0}^{l} {\omega \left( {q_{0} + \gamma_{2} h_{2} } \right)dx} = \int_{0}^{l} {\left[ {\frac{{\left( {q_{0} + \gamma_{2} h_{2} } \right)^{2} x^{2} }}{24EI}\left[ {\left( {6l^{2} + x^{2} - 4lx} \right) - 2l\left( {3l - x} \right)} \right]} \right]} dx$$

where *E* is the elastic modulus and *I* is the moment of inertia.

It can be seen from the mathematical expression of Formula (10) that a part of the released elastic energy is transmitted to the supporting coal body to form dynamic load stress, which is superimposed with the static supporting stress of the coal body to produce ‘ dynamic-static ‘ stress and reduce the stability of the coal pillar.

In general, the dynamic load stress *P*_*d*_ is about *k*_*d*_ times of the static load stress *P*, that is, *P*_*d*_ = *K*_*d*_*P*. Therefore, considering the average stress *P*_*z*_ borne by the interval pillar under dynamic-static loading conditions, the mathematical expression is as follows:

11 $$P_{z} = P + P_{d} = \left( {1 + K_{d} } \right)\left[ \begin{gathered} \gamma H + \frac{\gamma H}{B}\left( {\frac{{W_{2} }}{2} - h_{1} \cot \alpha } \right) + \hfill \\ \frac{{\left( {q_{0} + \gamma_{2} h_{2} } \right)\left( {l + l_{m} } \right)}}{2B} + \frac{{\gamma_{1} h_{1}^{2} \left( {\cot \beta + 3\cot \alpha } \right)}}{4B} \hfill \\ \end{gathered} \right]$$

The strength of the interval coal pillar is calculated according to the Bineuski formula^24^:

12 $$\sigma_{q} = \sigma_{j} \left( {0.64 + 0.54\frac{B}{M}} \right)$$

where *σ*_*j*_ is the uniaxial compressive strength of coal; *M* is the height of interval coal pillar.

In order to maintain the stability of the interval coal pillar, it is also necessary to consider the weakening effect of the coal body. Under the action of dynamic load stress and static load stress, the uniaxial compressive strength of the interval coal pillar is obviously reduced^25^.

Considering the reduction degree of the uniaxial compressive strength of the interval coal pillar, the mathematical expression for the critical width *B* of the interval coal pillar to maintain stability is:

13 $$\left( {1 + K_{d} } \right)\left[ \begin{gathered} \gamma H + \frac{\gamma H}{B}\left( {\frac{{W_{2} }}{2} - h_{1} \cot \alpha } \right) + \hfill \\ \frac{{\left( {q_{0} + \gamma_{2} h_{2} } \right)\left( {l + l_{m} } \right)}}{2B} + \frac{{\gamma_{1} h_{1}^{2} \left( {\cot \beta + 3\cot \alpha } \right)}}{4B} \hfill \\ \end{gathered} \right] \le k_{x} \sigma_{j} \left( {0.64 + 0.54\frac{B}{M}} \right)$$

where *k*_*x*_ is the reduction coefficient of the uniaxial compressive strength of coal pillar, with *k*_*x*_ ranging from 0.7 to 0.9.

**Fig.6:**
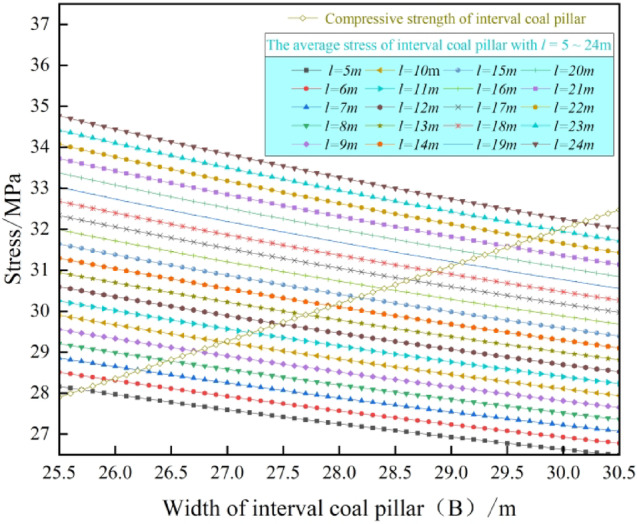
Interval coal pillar reasonable size of *l* = 5 ~ 24 m.

Substitute the calculated parameters of the surrounding rock of the 3# coal seam from Table 1 into Eq. (13), and the results are shown in Fig. 6.

**Table 1:** The engineering geological data.

*γ* = 2500 kg/m^3^	*σ*_*j*_ = 8.9 MPa	*h*_*2*_ = 24.6 m	*α* = 62°
*γ*_*1*_ = 2300 kg/m^3^	*l* = 5 m ~ 24 m	*W*_*2*_ = 4.4 m	*β* = 38°
*γ*_*2*_ = 2400 kg/m^3^	B = 25.5 m ~ 30.5 m	*M* = 4.2 m	*K*_*d*_ = 1.3
*q*_*0*_ = 7.09 MPa	*h*_*1*_ = 3.8 m	*l*_*m*_ = 24 m	*k*_*x*_ = 0.8

As shown in Fig. 6, the size of interval coal pillar *B* increases with the extension of the exposed length *l* of the basic roof above the main withdrawal channel. When *l* increases from 5 to 24 m, the reasonable size of the interval coal pillar increases from 25.8 to 30.2 m. Therefore, in the process of connecting the coal working mining face with the main withdrawal channel, the use of pressure adjusting technology or roof cutting technology to control the hanging length of the basic roof on the main withdrawal channel can shorten the size of the interval coal pillar and improve the recovery rate of the coal body.

**Fig. 7:**
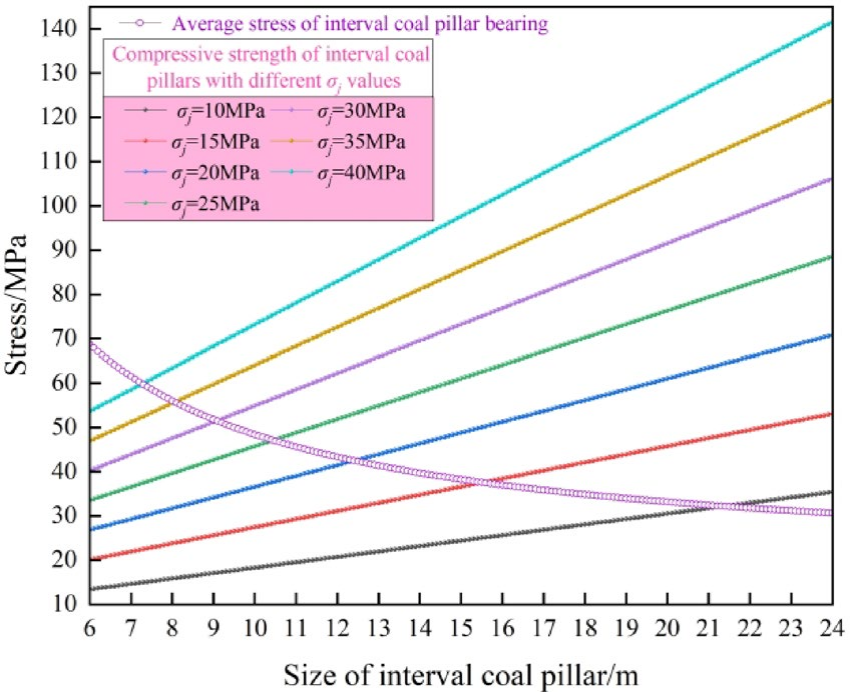
Reasonable size of interval coal pillar with different *σ*_*j*_ values.

When adopting reasonable pressure adjusting technology or roof cutting technology and considering the space required for retracement of coal mining equipment, the suspension length *l* of the overlying basic roof of the main withdrawal channel is 10 m, which is most conducive to controlling the stability of the surrounding rock of the main withdrawal channel , reducing the size of the interval coal pillar and improving the coal recovery rate. On this basis, increasing the strength of the interval coal pillar can further shorten the size of the interval coal pillar. Therefore, the parameters of *l* = 10 m, *σ*_*j*_ = 10 MPa ~ 40 MPa, *k*_*x*_ = 0.95, *B* = 6 m ~ 24 m and Table 1 are substituted into formula (13) to obtain the reasonable size of interval coal pillar with different strength as shown in Fig. 7.

As shown in Fig. 7, the reasonable size of interval coal pillar *B* decreases with the increase of uniaxial compressive strength *σ*_*j*_. When *σ*_*j*_ is enhanced from 10 to 40 MPa, the reasonable size of the interval coal pillar is reduced from 21.4 to 7.3 m, and the size of the interval coal pillar is reduced by 65.9%. It can be seen that enhancing the uniaxial compressive strength of the interval coal pillar can significantly shorten the size of the interval coal pillar and improve the recovery rate of coal.

## A technology of pre-excavated double withdrawal channel without coal pillars abandonment

### Traditional technology

**Fig. 8:**
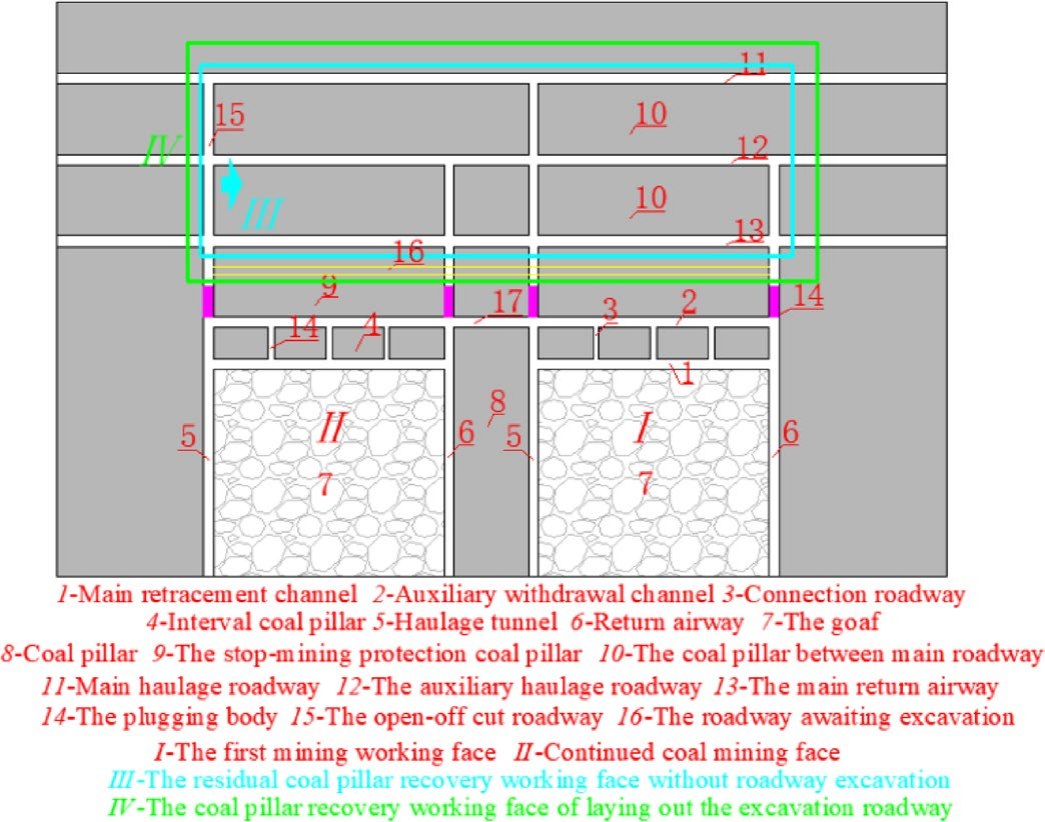
Traditional technology.

Traditional technologies of recovering residual coal pillars for pre-excavation double withdrawal channels are divided into non-roadway excavation for recovering residual coal pillars and layout of excavation roadway for recovering residual coal pillars, as shown in Fig. 8.

As shown in Fig. 8, traditional technology of recovering residual coal pillars for pre-excavation double withdrawal channel is to use the plugging body 14 to plug the transportation roadway 5 and the return air roadway 6 after the mining of the coal workinbg mining face. The open-off cut 15 of the coal pillar recovery working face *III* is arranged between the main transportation roadway 11 and the main return airway 13. The main transportation roadway 11 and the main return airway 13 are respectively used as the transportation roadway and the return air roadway of the coal pillar recovery working face *III*. Although this technology of recovering residual coal pillars without roadway excavation can minimize the workload and cost of roadway excavation, the stop-mining protective coal pillar 9 and the interval coal pillar 4 are abandoned, resulting in the waste of coal resources.

Similarly, after the mining of the coal working mining face is completed, the transportation roadway 5 and the return air roadway 6 are blocked with the plugging body 14. In the stop mining protection coal pillar 9, the roadway 16 is excavated as the return air roadway of the recovery residual coal pillar working face *IV*, and the open-off cut 15 of the recovery residual coal pillar working face *IV* is arranged between the roadway 16 and the main transportation roadway 11. Although most of the coal pillars are recovered by this excavation roadway technology, some of the protective coal pillar 9 and all the interval coal pillar 4 are abandoned, which also increases the workload and cost of roadway excavation.

The coal mine adopts the coal pillar recovery technology without roadway excavation. The width of interval coal pillar arranged during the stop-mining period of 3301 working face is 28 m. According to the theoretical calculation in Section "Project overview", after adopting the roof cutting technology, the width of interval coal pillar with the hanging length of *l* = 10 m on the main withdrawal channel is 26.8 m, the recovery rate of interval coal pillar is increased by 4.3%, and most interval coal pillars and stop-mining protection coal pillars are still abandoned, resulting in a large number of coal resource losses.

### Technology of no coal pillar abandonment around pre-excavation double withdrawal channel

**Fig. 9:**
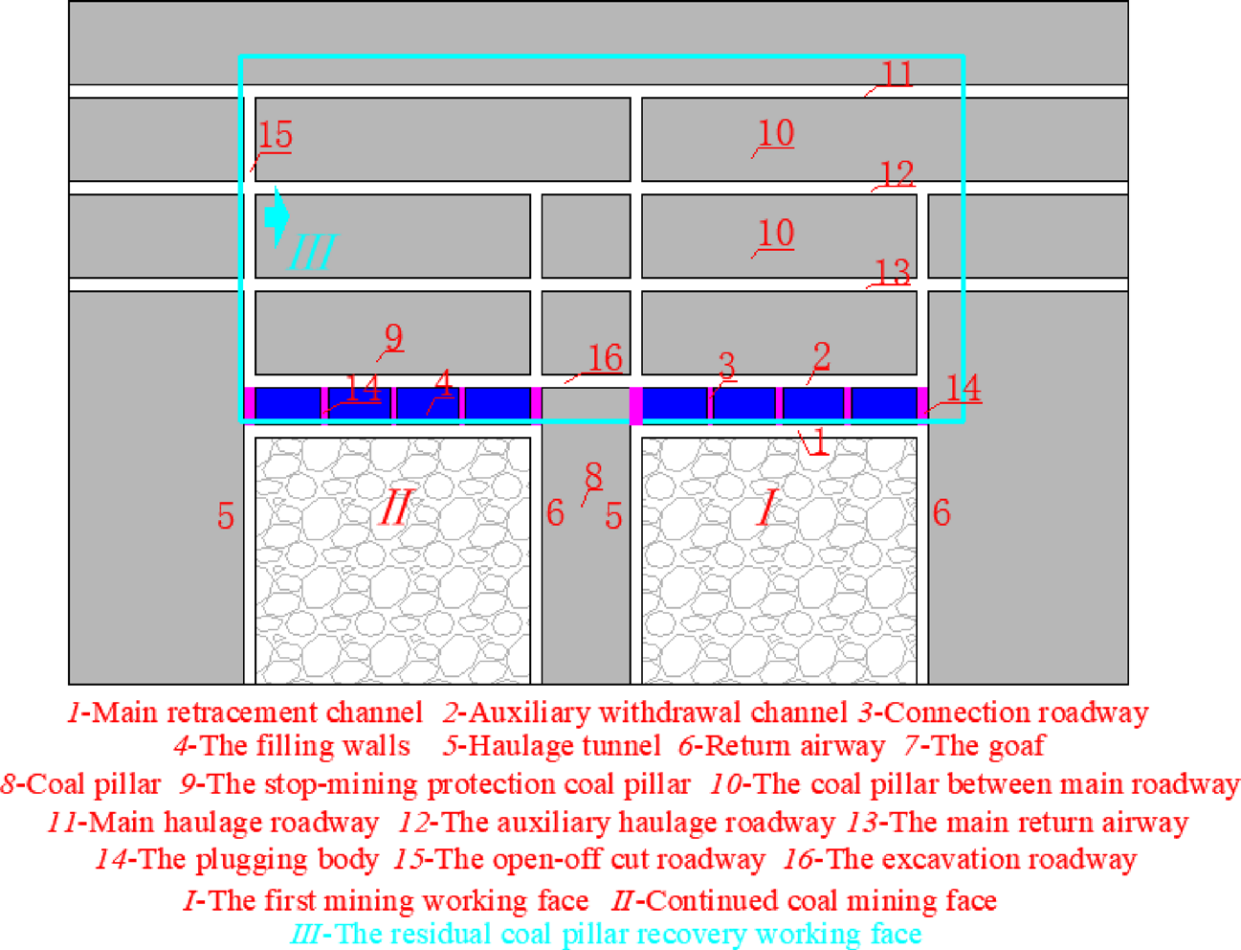
The technology of pre-excavation double-withdrawal channel without coal pillar abandonment.

From the theoretical calculation in section "Size setting of interval coal pillar", it can be seen that increasing the strength of the interval coal pillar can reduce the size of the interval coal pillar and improve the stability of the surrounding rock of the withdrawal channel. At the same time, combining the advantages and disadvantages of the 2 types of coal pillar recovery techniques in Section "Traditional technology", a technology of pre-excavation double-withdrawal channel without coal pillar abandonment is proposed, which can completely recover coal pillars in the surrounding rock of the pre-excavation double retraction channel and realize the maximum recovery rate of coal resources. This technology is shown in Fig. 9.

According to Fig. 9, step ① , before the first coal mining working face *I* enters the end mining period, the filling wall 4 is used to replace the interval coal pillar. Step ②, the equipment relocation work will be carried out normally in the stop mining stage. Step ③, after the first mining face Ⅰ moves the coal mining equipment, the return airway 6, haulage roadway 5 and contact roadway 3 are blocked with the plugging body 14*.* Step ④, the coal resources in the continuous working face *II* are mined, and steps ① ~ ③ are repeated. Step ⑤, the excavation roadway 16 is arranged between the haulage roadway 5 of the first mining face *I* and the return airway 6 of the continuous working face *II*. Step ⑥, the original auxiliary withdrawal channel 2 and the main haulage roadway 11 constitute the coal pillar recovery working face *III*, the main haulage roadway 11 is used as the transportation roadway of the coal pillar recovery working face *III*, and the original auxiliary withdrawal channel 2 and the excavation roadway 16 constitute the return airway of the coal pillar recovery working face *III*.

From Fig. 9, it can be seen that the technology of pre-excavation double-withdrawal channel without coal pillar abandonment can not only recover the interval coal pillar, the stop mining protection coal pillar and the coal pillar between the main roadways, but also not greatly increase the workload and cost of roadway excavation, which can perfectly solve the shortcomings of the traditional coal pillar recovery technology. The pre-excavation double retracement channel technology without coal pillar abandonment was formed, which broke through the traditional ‘ retracement channel excavation-equipment transfer-roadway abandonment ‘ mode, and realized the integrated process of’ retracement channel excavation-equipment transfer-reserving retracement channel-reserved retracement channel auxiliary production-retracement channel recovery coal pillar ‘ in the working face. In addition, Before the retained withdrawal channel is used as the roadway for recovering coal pillars, the retained retracement channel can also have functions such as storage equipment, storage materials, auxiliary transportation and gas collection.

### Numerical simulation study

#### The establishment of numerical simulation model

**Fig. 10:**
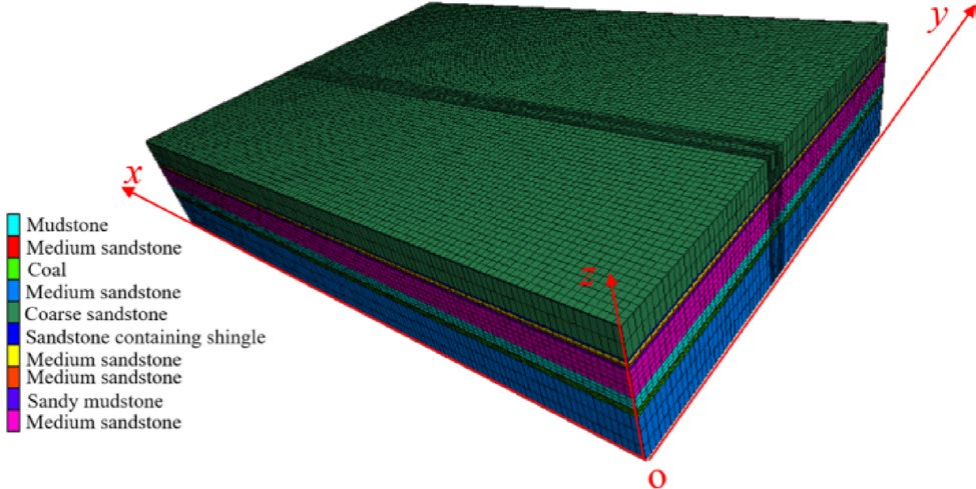
The pictures of the model.

According to the physical and mechanical parameters of the roof and floor of the *3#* coal seam in Table 2, the FLAC3D numerical model is established. The constitutive model is Mohr–Coulomb model. The bottom of the model is fixed, and the horizontal displacement constraints are applied to the two sides of the model and the front and rear boundaries.The model size is : *x* × *y* × *z* = 514 m × 520 m × 93 m, and 6.1 MPa is applied at the top of the model to simulate the load of the overlying strata. The width of the coal working mining face is 197.6 m. The model consists of 233,640 units. The numerical simulation model simulates the mining process of 3301 working face, 3302 working face and coal pillar recovery working face in 3# coal seam. Specifically, the 3301 working face is the first coal mining working face, 3302 working face is the continuous coal mining working face, and coal pillar recovery working face is the last mining working face. The model is shown in Fig. 10.

**Table. 2:** Physical and mechanical parameters of 3 # coal seam roof and floor strata.

Rock strata	Density (kg/m^3^)	Bulk modulus(GPa)	Shear modulus(GPa)	Cohesion (MPa)	Friction angle(°)	Tensile strength(MPa)
Coarse sandstone	2500	6.94	5.21	2.12	26	2.82
Sandstone containing shingle	2400	2.11	1.01	0.97	18	0.85
Sandy mudstone	2400	4.02	2.07	1.98	19	1.53
Medium sandstone	2500	8.16	5.62	2.31	27	2.98
Sandy mudstone	2400	4.02	2.07	1.98	19	0.85
Medium sandstone	2500	8.39	6.3	2.43	28	3.01
Mudstone	2300	2.04	0.83	0.85	18	0.6
Medium sandstone	2500	8.16	5.62	2.31	27	2.98
Coal	1440	1.3	0.7	1.3	25	0.6
Medium sandstone	2500	8.16	5.62	2.31	27	2.98
Filling wall	2460	4.88	3.81	3.3	40	1.5

According to Fig. 2 and the conclusion of section "A technology of pre-excavated double withdrawal channel without coal pillars abandonment" , the roadways are excavated in the model. The interval coal pillar between the main withdrawal channel and the auxiliary withdrawal channel is replaced by the filling wall with the uniaxial compressive strength of 40 MPa. The sizes of the filling wall are 7 m, 8 m, 9 m and 10 m respectively, and the roof is cut off at a position 6 m from the main withdrawal channel.

#### Simulation of reasonable filling wall size

**Fig. 11:**
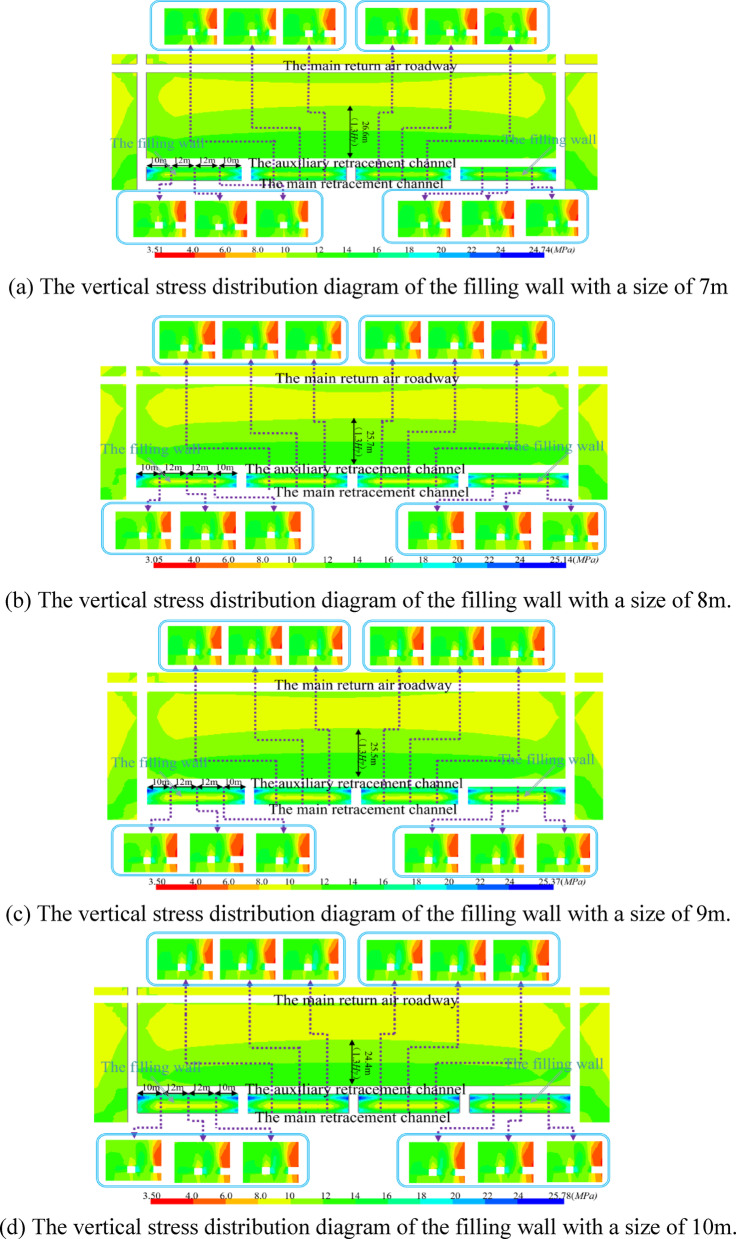
The vertical stress distribution diagram of the filling wall with a size of 7 m ~ 10 m.

The safe and efficient removal of equipment depends on the stability of the interval coal pillar. Therefore, before the mining influence of the coal mining working face spreads to the surrounding area of the withdrawal channel, the filling wall is used to replace the interval coal pillar. The width of the filling wall used in the first mining working face is 7 m ~ 10 m. After the working face is connected with the main withdrawal channel, the vertical distribution of the stop mining stage is shown in Fig. 11.

From Fig. 11, it can be seen that the four right-angled regions of the filled wall are stress-concentrated regions, and the degree of stress concentration tends to increase as the size of the filled wall increases. In the process of increasing the size of the filled wall from 7 to 10 m, the region of vertical stresses of 8 MPa to 10 MPa distributed within the filled wall widened continuously, indicating the increasing stability of the filled wall. During the stop mining period, with the increase of the size of the filling wall, the influence of the auxiliary withdrawal channel on direction of the main return air roadway is reduced from 26.6 to 24.4 m, and the distance between the main return air roadway and the auxiliary withdrawal channel is 44 m, so the surrounding rock of main return air roadway is basically unaffected. In summary, it shows that the size of the wall is greater than 7 m to ensure its needs.

**Fig. 12:**
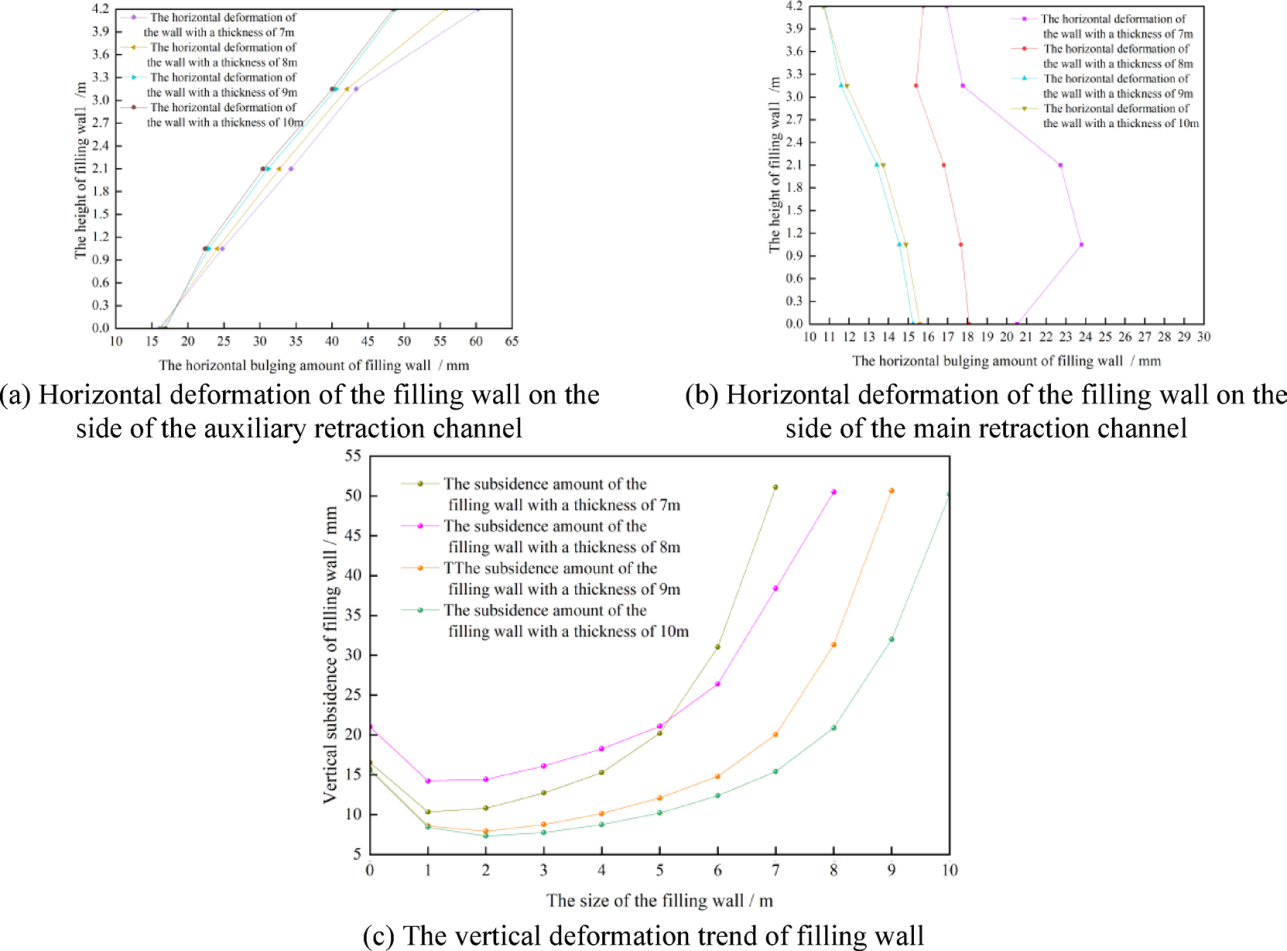
Deformation of filling wall.

Whether the bearing capacity of the filling wall meets the needs of the work of the migrating coal mining equipment can also be reflected by the vertical and horizontal deformation of the filling wall. The vertical and horizontal deformation trends of the filling wall with different sizes are shown in Fig. 12.

It can be seen from Fig. 12 that with the increase of the size of the filling wall, the deformation of the filling wall tends to decrease. The horizontal and vertical deformations on the auxiliary withdrawal channel side of the filled wall are both greater than those on the main withdrawal channel side of the filled wall. This is because under the movement trend of fracture, rotation and subsidence of overlying strata, a certain range of stress adjustment area is formed in front of coal mining working face. During the rotational and sinking movements of the already fractured rock blocks, a downward inclined thrust *P* is formed in front of the goaf.

**Fig. 13:**
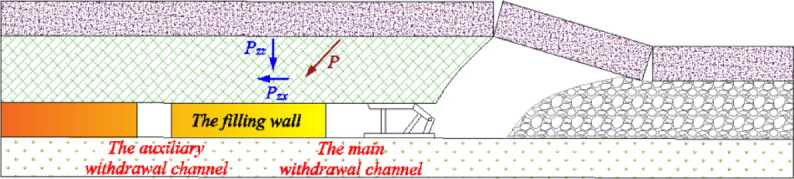
The stress distribution diagram in front of the stop mining work face.

When the coal mining working face is connected to the main withdrawal channel, under the action of thrust *P*, the stress field between the auxiliary withdrawal channel and the main withdrawal channel will change. At the same time, the stress field around the filling wall has shear stress *P*_*zx*_ and vertical stress *P*_*zz*_, as shown in Fig. 13. Therefore, under the action of shear stress *P*_*zx*_, vertical stress *P*_*zz*_ and thrust *P*, the horizontal and vertical deformations on the auxiliary withdrawal channel side of the filled wall are both greater than those on the main withdrawal channel side of the filled wall. As the filling wall size increased from 7 to 10 m, the maximum horizontal deformation on the main withdrawal channel side, the maximum horizontal deformation on the auxiliary withdrawal channel side, and the maximum top subsidence of the filling wall are 23.8 mm ~ 15.6 mm, 60.3 mm ~ 48.5 mm, and 51.1 mm ~ 50.2 mm, respectively.

#### Simulation study on recovery of remaining coal pillars

After the removal of the coal mining equipment from the first mining face is completed, the connecting roadway, return airway and transportation roadway of the first mining face are sealed, and the same technology as that used for the first mining face is adopted to mine the successor working face. Similarly, after the removal of the coal mining equipment from the continuous working face is completed, the connecting roadway, return airway and transportation roadway should be sealed. A roadway is excavated between the auxiliary withdrawal channel of the first coal mining working face and the auxiliary withdrawal channel of the continuation coal mining working face. The auxiliary withdrawal channel of the first coal mining working face, the auxiliary withdrawal channel of the continuation coal mining working face and the newly excavated roadway together form the return airway of the residual coal pillar recovery working face. The main haulage roadway is used as the transportation roadway of the residual coal pillar recovery working face.

**Fig. 14:**
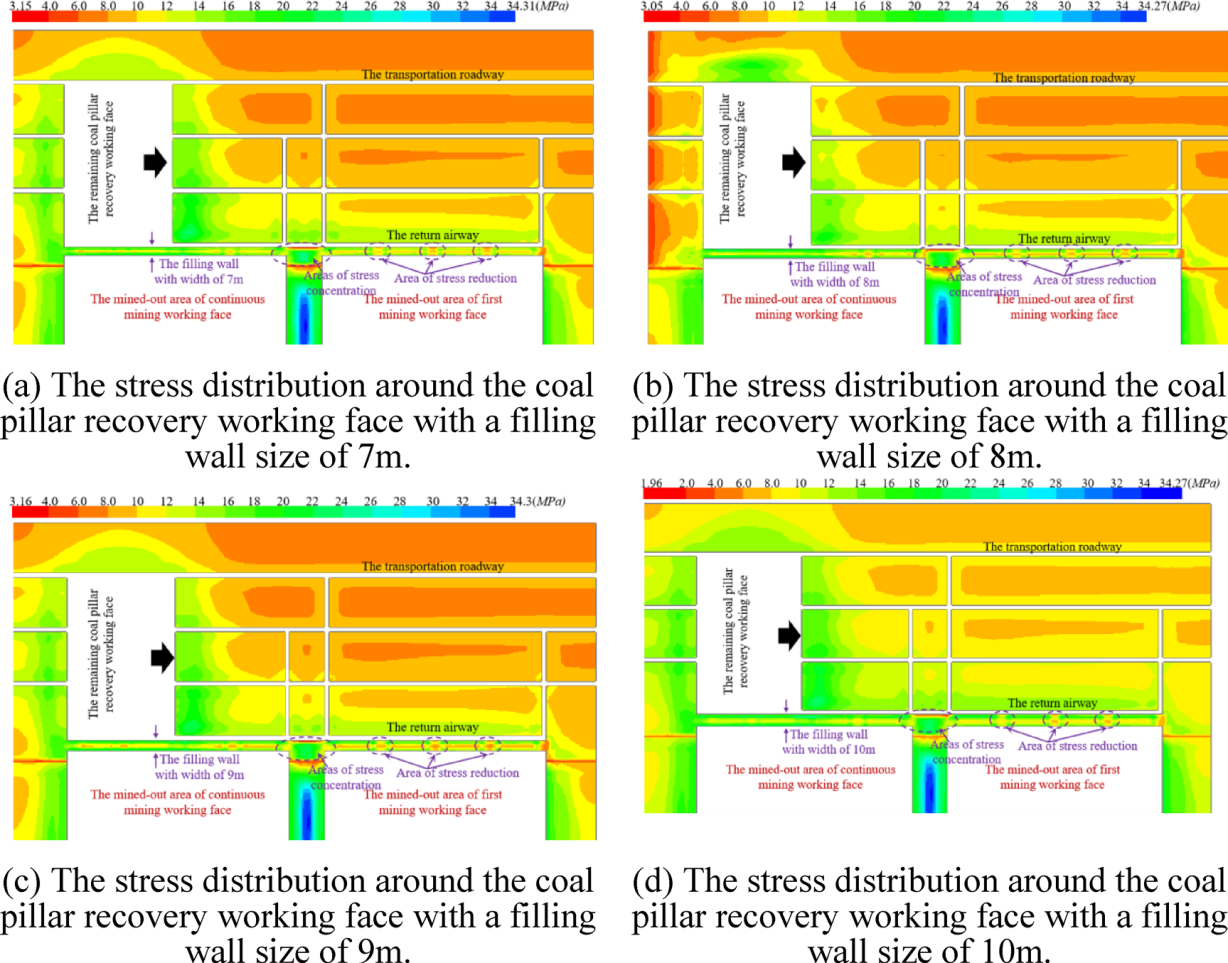
The vertical stress distribution around the remaining coal pillar recovery working face with the filling wall size of 7 m ~ 10 m.

The mining of the remaining coal pillar recovery working face is simulated. When the size of the filling wall is 7 m ~ 10 m, the vertical stress distribution around the remaining coal pillar recovery working face is shown in Fig. 14.

The size of the filling wall is 7 m ~ 10 m, which has a good supporting capacity, and the vertical stress around the return aiway is greater than the vertical stress around the transportation roadway. Moreover, as the width of the filling wall increases, the mining influence of the coal pillar recovery working face on the area of the vertical stress redistribution within the filling wall becomes smaller.

**Fig. 15:**
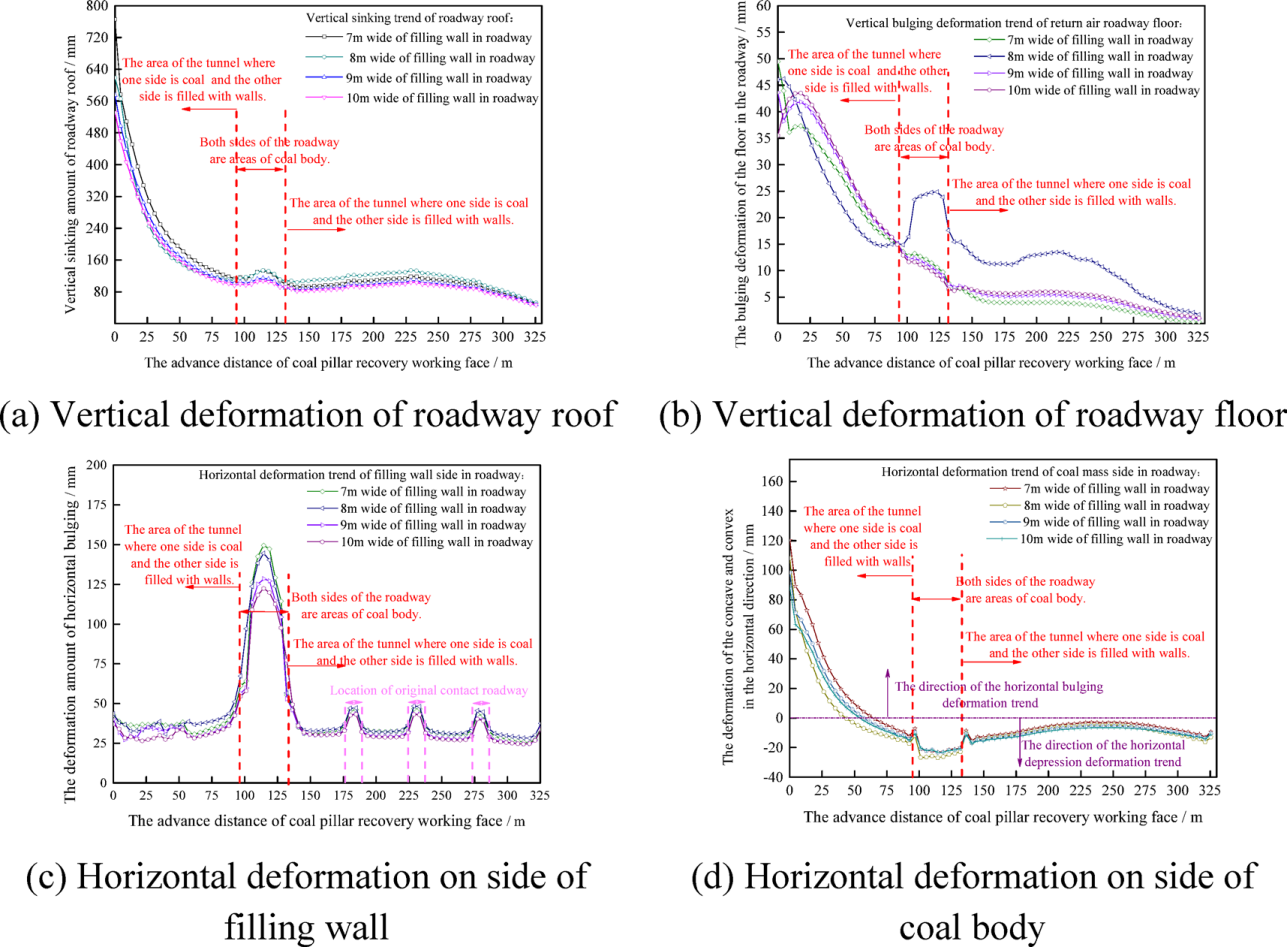
The surrounding rock deformation of the return airway in coal pillar recovery working face.

Stress concentration occurs in the roadway between the auxiliary withdrawal channel of the first coal mining working face and the auxiliary withdrawal channel of the continuation coal mining working face, and special attention should be paid to the deformation of roadway in this area. The filling wall in the contact roadway area is in the low stress area. However, the roadway protected by the filling wall needs to be retained for a long time to serve the coal pillar recovery working face. Under the mining influence of the coal pillar recovery working face, the surrounding rock deformation of the return airway ( the roadway protected by the filling wall ) is shown in Fig. 15.

It can be seen from Fig. 15a that with the increase of the distance of the advanced coal mining working face, the roof subsidence of the return airway is basically decreasing. When the widths of the filling walls are 7 m, 8 m, 9 m and 10 m respectively, the vertical subsidence of the roadway roof is 765.6 mm, 618.6 mm, 574.8 mm and 532 mm respectively at the 0 m position of the advanced coal pillar recovery working face, and the vertical subsidence of the roadway roof is 52.6 mm, 53.5 mm, 48.3 mm and 46.7 mm respectively at the 325 m position of the advanced coal pillar recovery working face. It can be seen from Fig. 15b that with the increase of the distance of the advanced coal mining working face, the floor deformation of the return airway is a downward trend of fluctuation. When the widths of the filling walls are 7 m, 8 m, 9 m and 10 m respectively, the vertical bulge of the roadway floor is 49.5 mm, 46 mm, 43.6 mm and 35.5 mm respectively at the 0 m position of the advanced coal pillar recovery working face, and the vertical bulge of the roadway floor is 1.4 mm, 1.7 mm, 1.9 mm and 1.1 mm respectively at the 325 m position of the advanced coal pillar recovery working face. It can be seen from Fig. 15c that with the increase of the distance of the advanced coal mining working face, the horizontal deformation on the side of the filling wall in the roadway is a fluctuating form. When the widths of the filling walls are 7 m, 8 m, 9 m and 10 m respectively, the horizontal bulging amounts of the filled walls are 43.7 mm, 43.5 mm, *3*9.9 mm and 36.9 mm respectively at the 0 m position of the advanced coal pillar recovery working face, and horizontal bulging amounts of the filled walls are 34.4 mm, 34.5 mm, 33.8 mm and 33.6 mm respectively at the 325 m position of the advanced coal pillar recovery working face. It can be seen from Fig. 15d that with the increase of the distance of the advanced coal mining working face, the deformation on one side of the coal body in the roadway shows a trend of first horizontal protrusion deformation and then horizontal depression deformation. When the widths of the filling walls are 7 m, 8 m, 9 m and 10 m respectively, the deformation of the horizontal bulge on one side of the coal body is 120.2 mm, 107.9 mm, 98.4 mm and 88.6 mm respectively at the 0 m position of the advanced coal pillar recovery working face, and the deformation of the horizontal depression on one side of the coal body is 9.3 mm, 12.9 mm, 10.5 mm and 11 mm respectively at the 325 m position of the advanced coal pillar recovery working face.

Considering the mining process of the first coal mining working face, the continuous coal mining working and the coal pillar recovery working face, and considering the cost of the filling wall, the deformation of the surrounding rock of the roadway, the stress distribution of the surrounding rock of the roadway and the safety of the long-term retention of the roadway, the reasonable size of the filling wall is 8 m.

## Supporting structure of roadway surrounding rock

### Study on surrounding rock support of main withdrawal channel

#### Unstable failure of residual coal body

**Fig. 16:**
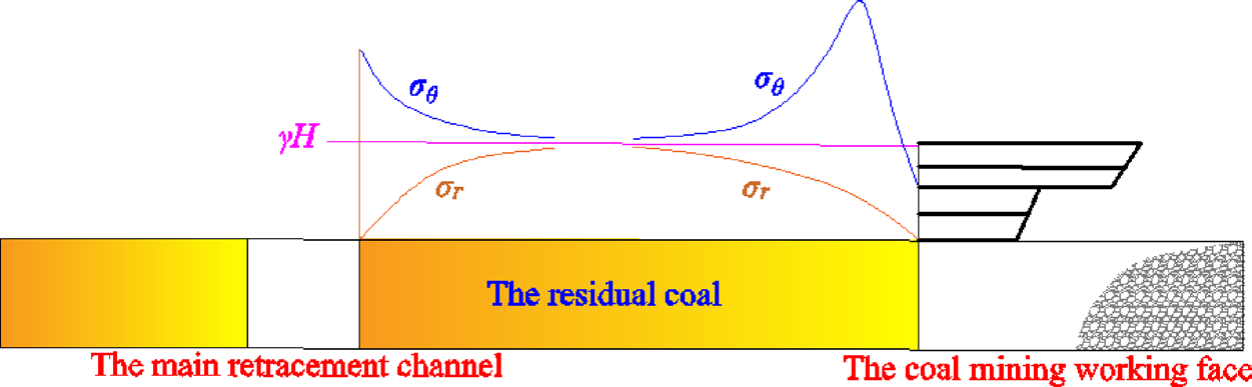
The stress distribution between the main withdrawal channel and the coal mining working face.

Before studying the surrounding rock support parameters of the main withdrawal channel, it is necessary to study the instability size of the residual coal body between the coal mining working face and the main withdrawal channel. When the coal mining working face is far away from the main withdrawal channel, the advance support stress of the coal mining working face will not affect the surrounding rock of the main withdrawal channel. The the surrounding rock of the main withdrawal channel is only affected by the section size of the main withdrawal channel and the stress of the surrounding rock. The main withdrawal channel can be regarded as the shape of a circular hole, and the coal mining working face can also be regarded as the shape of a circular hole. Further, the stress distribution of two adjacent holes with unequal diameters between the main withdrawal channel and the coal mining working face under different spacing conditions is studied. *σ*_*θ*_ is the tangential stress around the circular hole, *σ*_*r*_ is the radial stress around the circular hole, and *γH* is the vertical load above the circular hole, as shown in Fig. 16.

**Fig. 17:**
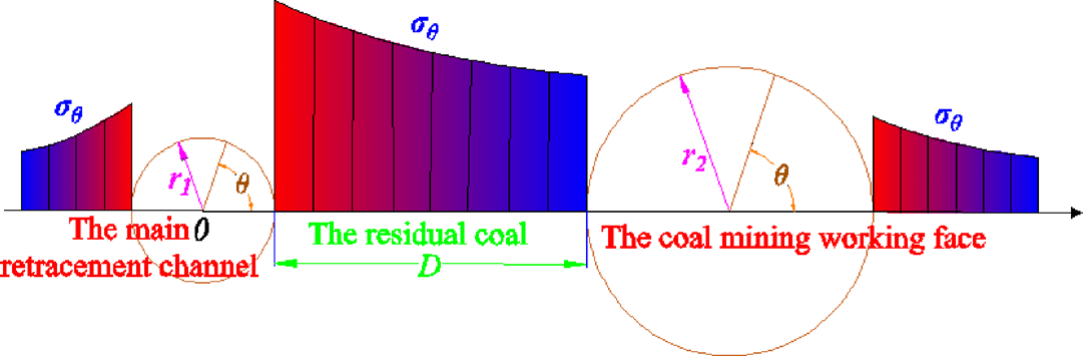
Stress superposition model of unequal diameter adjacent two holes.

In order to further analyze the mechanical structure of Fig. 16, the height of the main withdrawal channel is taken as the radius *r*_*1*_ of the circle, and the height of the caving zone of the coal mining working face is taken as the radius *r*_*2*_ of the circle. The size of the residual coal body between the main withdrawal channel and the coal mining working face is defined as* D*. When the advanced abutment stress of the coal mining working face spreads to the surrounding rock of the main withdrawal channel, the stress superposition model between the main withdrawal channel and the coal mining working face as shown in Fig. 17 can be established. The stress distribution between the two holes can be solved by elastic analysis method. The vertical stress of the residual coal body between the main withdrawal channel and the coal mining working face is the tangential stress with the center angle of *θ* = *0°*, and the center coordinate of the main withdrawal channel is the origin.

In the residual coal body, according to the elastic analysis method, the vertical stresses on the side of the main withdrawal channel and the side of the coal mining working face are :

14 $$\left\{ \begin{gathered} \sigma_{\theta 1} = \frac{\gamma H}{2}\left( {1 + \frac{{r_{1}^{2} }}{{x_{1}^{2} }}} \right) + \frac{\gamma H}{2}\left( {1 + 3\frac{{r_{1}^{4} }}{{x_{1}^{4} }}} \right) \hfill \\ \sigma_{\theta 2} = \frac{\gamma H}{2}\left[ {1 + \frac{{r_{2}^{2} }}{{\left( {r_{1} + r_{2} + D - x_{1} } \right)^{2} }}} \right] + \frac{\gamma H}{2}\left[ {1 + 3\frac{{r_{2}^{4} }}{{\left( {r_{1} + r_{2} + D - x_{1} } \right)^{4} }}} \right] \hfill \\ \end{gathered} \right.\begin{array}{*{20}c} {} \\ \end{array} \left( {r_{1} \le x_{1} \le r_{1} + D} \right)$$

where *σ*_*θ1*_ is the concentrated stress generated by the main withdrawal channel in the residual coal body . *σ*_*θ2*_ is the concentrated stress generated in front of the coal mining face . *H* is the buried depth of coal seam . *γ* is the average density of rock strata.

The superimposed stress in the residual coal body is :

15 $$\sigma_{\theta } = \sigma_{\theta 1} + \sigma_{\theta 2} - \gamma H$$

Substituting formula (14) into formula (15), we get :

16 $$\sigma_{\theta } { = }\left[ \begin{gathered} \frac{{3\gamma Hr_{1}^{4} }}{{2x_{1}^{4} }} + \frac{{3\gamma Hr_{2}^{4} }}{{2\left( {r_{1} + r_{2} + D - x_{1} } \right)^{4} }} + \hfill \\ \frac{{\gamma Hr_{1}^{2} }}{{2x_{1}^{2} }} + \frac{{\gamma Hr_{2}^{2} }}{{2\left( {r_{1} + r_{2} + D - x_{1} } \right)^{2} }} + \gamma H \hfill \\ \end{gathered} \right]\begin{array}{*{20}c} {} & {\begin{array}{*{20}c} {} & {(r_{1} \le x_{1} } \\ \end{array} \le r_{1} + D)} \\ \end{array}$$

17 $$r_{2} = \frac{100M}{{4.7M + 19}} + 2.2$$

where *M* is the height mining of coal seam.

It can be seen from formula (16) that the degree of stress superposition in the residual coal body is related to the buried depth, the radius of the circular hole, the size of the residual coal body and other factors.

**Fig. 18:**
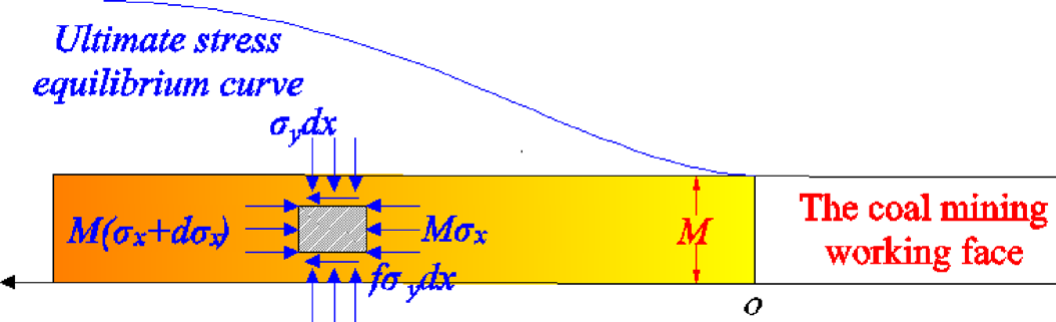
Mechanical model of residual coal body.

When the distance between the coal mining working face and the main withdrawal channel is far, the stress model of the residual coal body between the coal mining working face and the main withdrawal channel is shown in Fig. 18.

**Fig. 19:**
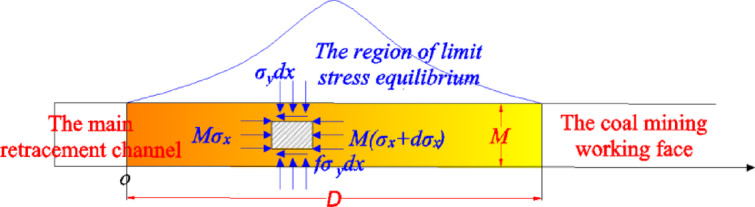
Ultimate stress model of residual coal body.

When the distance between the coal mining working face and the main withdrawal channel is close, that is, the residual coal body enters the limit stress equilibrium state as a whole, and the stress distribution of the residual coal body between the coal mining working face and the main withdrawal channel is shown in Fig. 19.

For the residual coal body between the main withdrawal channel and the coal mining working face, the limit equilibrium stress expression is :

18 $$\left\{ \begin{gathered} \sigma_{y} = nNe^{{\frac{{2x_{2} f\left( {1 + \sin \varphi } \right)}}{M(1 - \sin \varphi )}}} \begin{array}{*{20}c} {} & {(0 \le x_{2} \le {D \mathord{\left/ {\vphantom {D {2)}}} \right. \kern-0pt} {2)}}} \\ \end{array} \hfill \\ \sigma_{y} = nNe^{{\frac{{2f\left( {1 + \sin \varphi } \right)\left( {D - x_{2} } \right)}}{M(1 - \sin \varphi )}}} \begin{array}{*{20}c} {} & {{{(D} \mathord{\left/ {\vphantom {{(D} 2}} \right. \kern-0pt} 2} \le x_{2} \le D)} \\ \end{array} \hfill \\ \end{gathered} \right.$$

where *σ*_*y*_ is the vertical stress. *φ* is the internal friction angle. *N* is the compressive strength of the residual coal. *f* is the friction coefficient. *n* is the damage coefficient of the remaining coal, 0.7 ~ 0.9.

From formulas (14) to (18), it can be seen that the width of the residual coal body in the critical failure instability state is :

19 $$\left\{ \begin{gathered} \left[ \begin{gathered} \frac{{3\gamma Hr_{1}^{4} }}{{2x_{1}^{4} }} + \frac{{3\gamma Hr_{2}^{4} }}{{2\left( {r_{1} + r_{2} + D - x_{1} } \right)^{4} }} + \hfill \\ \frac{{\gamma Hr_{1}^{2} }}{{2x_{1}^{2} }} + \frac{{\gamma Hr_{2}^{2} }}{{2\left( {r_{1} + r_{2} + D - x_{1} } \right)^{2} }} + \gamma H \hfill \\ \end{gathered} \right] \ge nNe^{{\frac{{2x_{2} f\left( {1 + \sin \varphi } \right)}}{M(1 - \sin \varphi )}}} \begin{array}{*{20}c} {} & {(r_{1} \le x_{1} \le r_{1} + D,0 \le x_{2} \le {D \mathord{\left/ {\vphantom {D 2}} \right. \kern-0pt} 2})} \\ \end{array} \hfill \\ \left[ \begin{gathered} \frac{{3\gamma Hr_{1}^{4} }}{{2x_{1}^{4} }} + \frac{{3\gamma Hr_{2}^{4} }}{{2\left( {r_{1} + r_{2} + D - x_{1} } \right)^{4} }} + \hfill \\ \frac{{\gamma Hr_{1}^{2} }}{{2x_{1}^{2} }} + \frac{{\gamma Hr_{2}^{2} }}{{2\left( {r_{1} + r_{2} + D - x_{1} } \right)^{2} }} + \gamma H \hfill \\ \end{gathered} \right] \ge nNe^{{\frac{{2f\left( {1 + \sin \varphi } \right)\left( {D - x_{2} } \right)}}{M(1 - \sin \varphi )}}} \begin{array}{*{20}c} {} & {{{(r_{1} \le x_{1} \le r_{1} + D,D} \mathord{\left/ {\vphantom {{(r_{1} \le x_{1} \le r_{1} + D,D} 2}} \right. \kern-0pt} 2} \le x_{2} \le D)} \\ \end{array} \hfill \\ \end{gathered} \right.$$

**Fig. 20:**
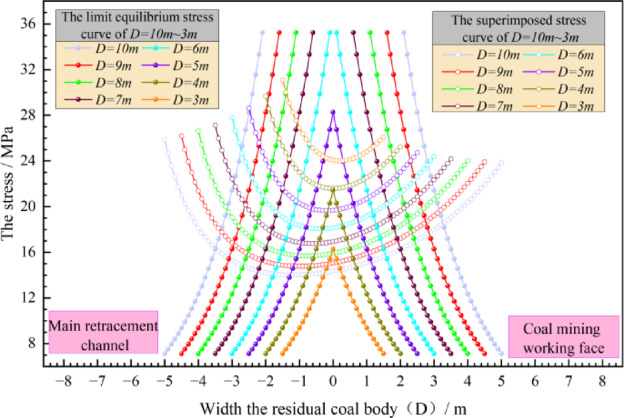
Limit width of residual coal body.

*H* = 312 m, *r*_*1*_ = 4.2 m, *M* = 4.2 m, D = 10 ~ 3 m, *n* = 0.8, *N* = 8.9, *f* = 0.47, *γ* = 2500 kg/m^3^, *φ* = 25° are brought into Eq. (19) as shown in Fig. 20.

**Fig. 21:**
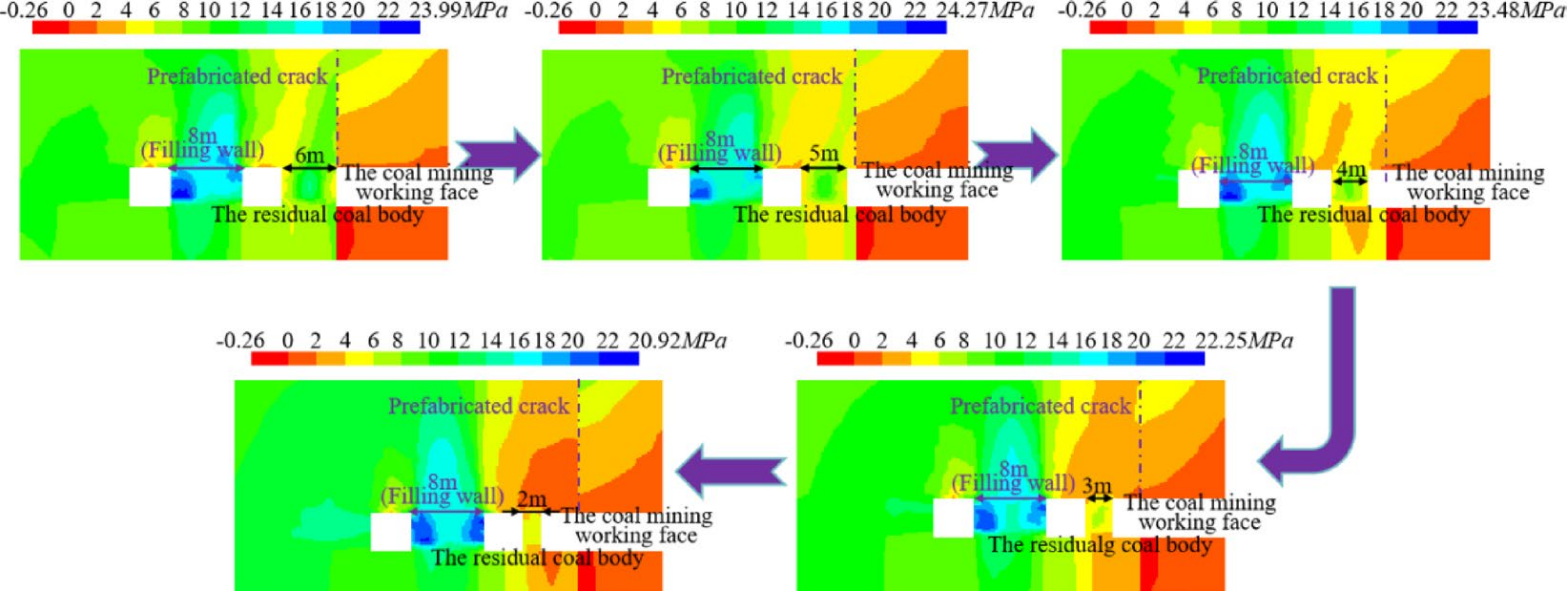
Vertical stress distribution of surrounding rock in main withdrawal channel.

It can be seen from Fig. 20 that with the reduction of the size of the residual coal body, the superimposed stress distributed in the residual coal body is increasing. When *r*_*1*_ and *M* are 4.2 m, the limit size of the residual coal body is 4 m. According to the conclusion of section "Size setting of interval coal pillar", the reasonable size of the filling wall is 8 m. The process of reducing the distance between the coal mining working face and the main withdrawal channel from 6 to 2 m is simulated, that is, the size of the residual coal body is reduced from 6 to 2 m. The vertical stress distribution of the surrounding rock of the main withdrawal channel is shown in Fig. 21.

As shown in Fig. 21, the peak values of vertical stresses within the residual coal body during the reduction of the residual coal body size from 6 to 2 m are 14.35 MPa, 13.23 MPa, 9.28 MPa, 6.28 MPa and 4.83 MPa, respectively. According to the vertical stress in the residual coal body of the above different sizes, when the size of the residual coal body is reduced from 4 to 3 m, the vertical stress in the residual coal body is lower than the original rock stress of 7.5 MPa, indicating that the residual coal body is unstable. Therefore, the upper limit of the support range of the residual coal body side of the main withdrawal channel is between 3 m ~ 4 m.

#### The deviatoric stress distribution of surrounding rock

The main withdrawal channel is affected by the mining of the coal mining working face, so the support technology of the surrounding rock of the main withdrawal channel needs to induce the deviatoric stress^26^. In elastic–plastic mechanics, stress tensor is divided into spherical stress tensor and deviatoric stress tensor. When the stress state of a certain point in the rock mass is represented by the principal stress *σ*_*i*_, *σ*_*i*_ is divided into three mutually perpendicular principal stresses.

20 $$\left( {\begin{array}{*{20}c} {\begin{array}{*{20}c} {\begin{array}{*{20}c} {\begin{array}{*{20}c} {\sigma_{1} } \\ 0 \\ \end{array} } \\ 0 \\ \end{array} } & {\begin{array}{*{20}c} 0 \\ {\sigma_{2} } \\ 0 \\ \end{array} } \\ \end{array} } & {\begin{array}{*{20}c} 0 \\ 0 \\ {\sigma_{3} } \\ \end{array} } \\ \end{array} } \right){ = }\left( {\begin{array}{*{20}c} {\begin{array}{*{20}c} {\begin{array}{*{20}c} {\begin{array}{*{20}c} {\sigma_{{\mathrm{m}}} } \\ 0 \\ \end{array} } \\ 0 \\ \end{array} } & {\begin{array}{*{20}c} 0 \\ {\sigma_{{\mathrm{m}}} } \\ 0 \\ \end{array} } \\ \end{array} } & {\begin{array}{*{20}c} 0 \\ 0 \\ {\sigma_{{\mathrm{m}}} } \\ \end{array} } \\ \end{array} } \right) + \left( {\begin{array}{*{20}c} {\begin{array}{*{20}c} {\begin{array}{*{20}c} {\begin{array}{*{20}c} {\sigma_{1} - \sigma_{{\mathrm{m}}} } \\ 0 \\ \end{array} } \\ 0 \\ \end{array} } & {\begin{array}{*{20}c} 0 \\ {\sigma_{2} - \sigma_{{\mathrm{m}}} } \\ 0 \\ \end{array} } \\ \end{array} } & {\begin{array}{*{20}c} 0 \\ 0 \\ {\sigma_{3} - \sigma_{{\mathrm{m}}} } \\ \end{array} } \\ \end{array} } \right)$$

In the formula (20), the first term on the right is the spherical stress tensor (*σ*_*m*_), which causes the volume change of the rock mass unit. The second term on the right side is three deviatoric stress tensors, which cause the fracture of rock mass elements, where *σ*_*1*_-*σ*_*m*_ is the maximum principal stress tensor. In this paper, the maximum deviatoric stress is used to analyze the failure characteristics and evolution of roadway surrounding rock.

**Fig. 22:**
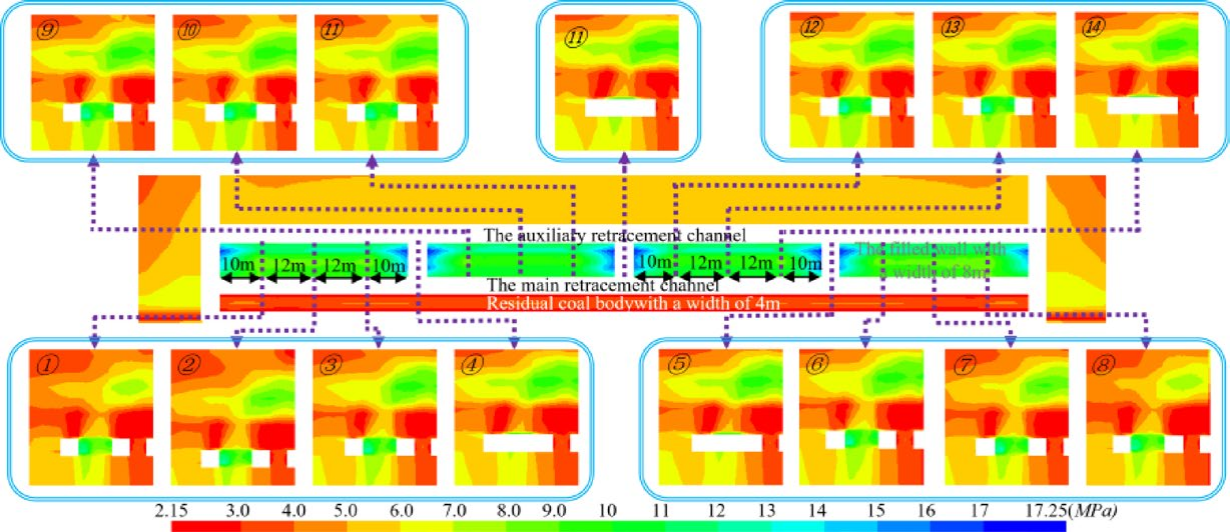
The deviatoric stress cloud diagram of the end mining stage of the working face.

It can be seen from section "a technology of pre-excavated double withdrawal channel without coal pillars abandonment" and "size setting of interval coal pillar" that the minimum size of residual coal bidy with bearing capacity is about 4 m during the end mining period of the working face, and the rational size of the filling wall is 8 m. Therefore, when the size of the residual coal body in the end mining stage of the coal mining working face is simulated to be 4 m, the deviatoric stress distribution is shown in Fig. 22.

**Fig. 23:**
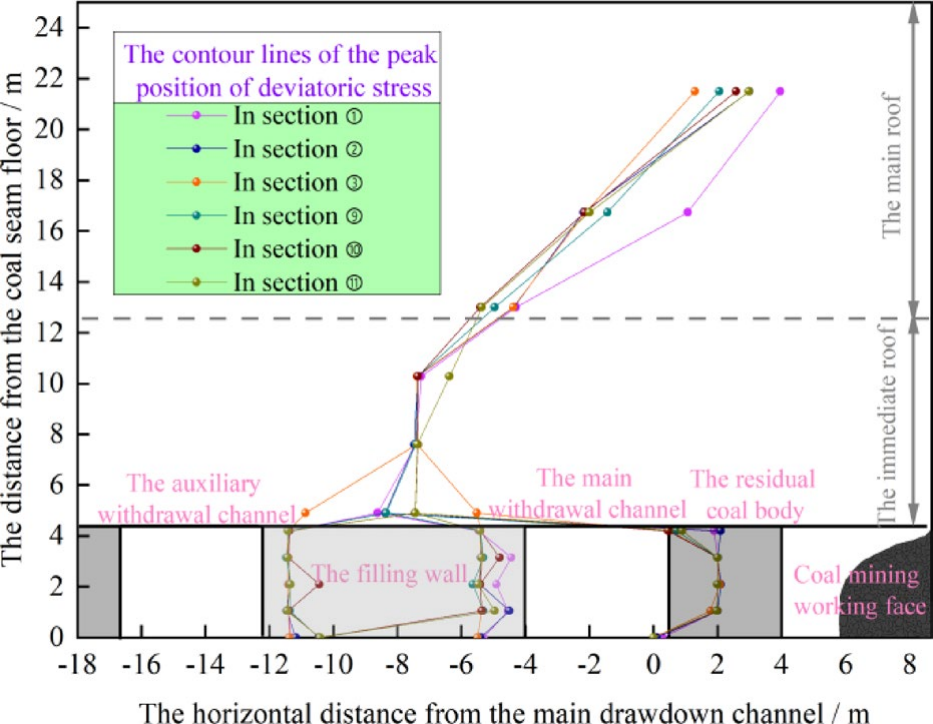
The contour lines of the peak position of deviatoric stress in sections ① ~ ③ and ⑨ ~ ⑪

As shown in Fig. 22, the distance between the coal mining working face and the main withdrawal channel is 4 m, that is, when the size of the residual coal body is 4 m, the deviatoric stress on both sides of the main withdrawal channel is greater than the original rock deviatoric stress of 3.2 MPa. The location of the peak deviatoric stress in the surrounding rock of the main retreat channel is more complicated, and the peak location of the deviatoric stress is mainly distributed in the center of the residual coal body, the edges on both sides of the filling wall and the overlying rock layer. Therefore, the contour lines of the peak position of deviatoric stress in sections ① ~ ③ and ⑨ ~ ⑪ are taken, as shown in Fig. 23.

From Fig. 23, it can be seen that on the side of the residual coal body in the main withdrawal channel, the horizontal distance from the peak contour line of the deviatoric stress to the main withdrawal channel is approximately 0.1 m to 2.1 m. Therefore, the lower limit of the support range on the side of the residual coal body is 2.1 m. The distance from the peak contour line of the deviatoric stress on one side of the filling wall to the main withdrawal channel is approximately 0.05 m to 6.06 m, and the lower limit of the support range on the filling wall side is 6.06 m. In the auxiliary withdrawal channel, the horizontal distance from the peak contour line of the deviatoric stress on the side of the filling wall to the auxiliary withdrawal channel is 0.95 m to 1.98 m, and the lower limit of the support range is 1.98 m. In the vertical direction, with the increase of the vertical distance from the withdrawal channel, the peak contour line of the deviatori stress gradually shifts to the side of the coal mining working face. Moreover, the vertical distance from the contour line of the peak deviatori stress to the roof of the withdrawal channel is approximately 11.7 m to 15.4 m, and the lower limit of the support is 11.7 m.

#### Supporting parameters of surrounding rock

**Fig. 24:**
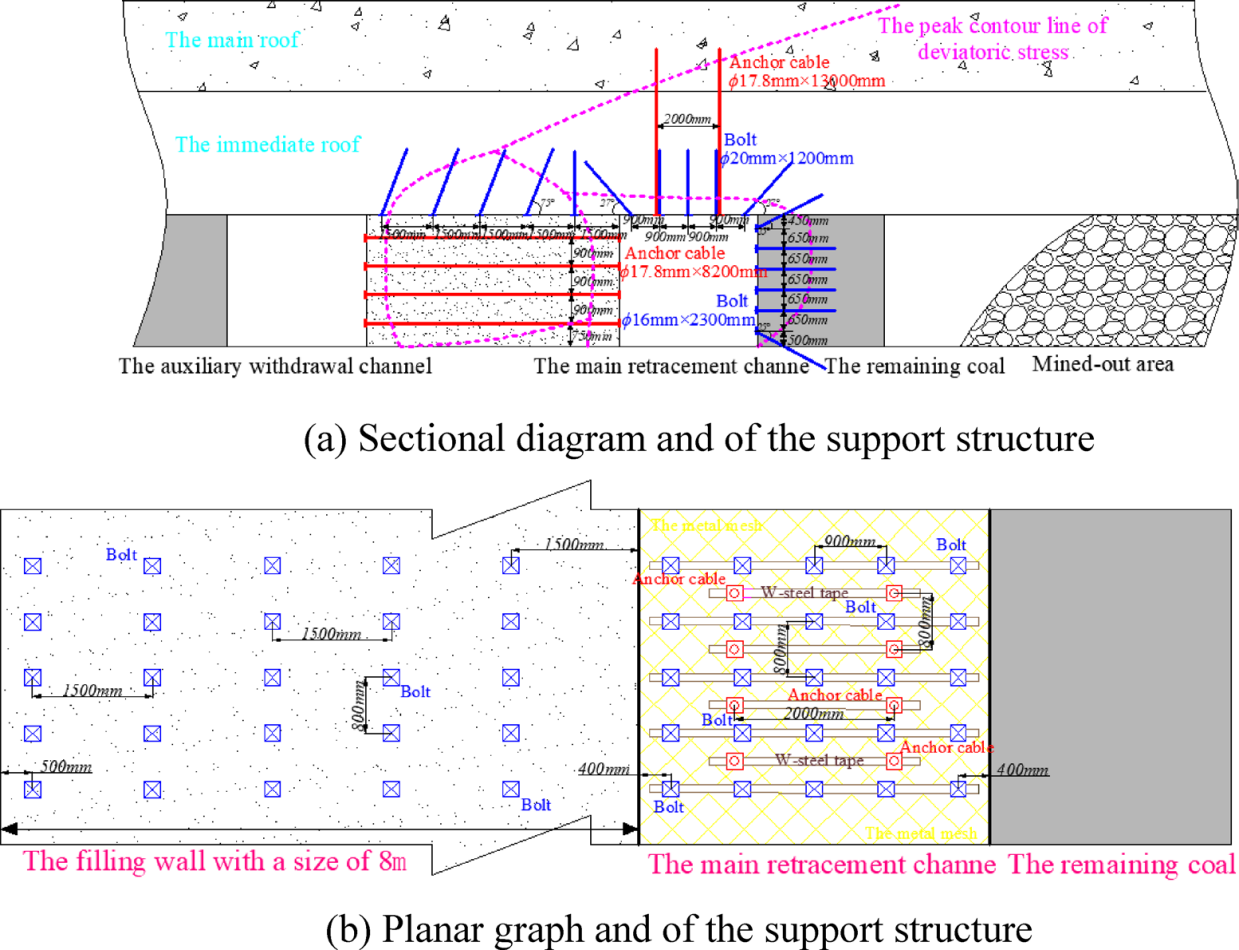
The supporting structure diagram of the main withdrawal channel.

Based on the peak contour line of the deviatoric stress of the surrounding rock in the main withdrawal channel, the roof of the main withdrawal channel is arranged with diamond wire mesh and W-shaped steel strip for combined support. The sectional diagram and planar graph of the support structure of surrounding rock supporting structure of main withdrawal channel are shown in Fig. 24. The parameters of the surrounding rock support structure of the main withdrawal channel are shown in Table 3.

**Table. 3:** Parameters of supporting structure.

The position	Parameters of supporting structure
The area of the roof	The length of the five φ20mm rebar bolts is 1200 mm, the spacing between the bolts is 900 mm, and the row spacing between the bolts is 800 mm 2. Two φ17.8 mm high pre-tightening force steel cable anchor cables are designed. The length of the anchor cable is 13000 mm, the spacing between the anchor cables is 2000 mm, and the row spacing between the anchor cables is 800 mm 3. The interval arrangement between anchor cable and anchor bolt is adopted
The area on the side of the residual coal body	The length of the five φ16mm reinforced fiber glass bolts is 2300 mm, the spacing between the bolts is 650 mm, and the row spacing between the bolts is 800 mm
Filling wall roof area	The length of the five φ20mm rebar bolts is 1500 mm, the spacing between the bolts is 1500 mm, and the row spacing between the bolts is 800 mm
The area of filling wall side	Four φ17.8 mm steel cable anchor cables are designed. The length of the anchor cable is 82,000 mm, the spacing between the anchor cables is 900 mm, and the row spacing between the anchor cables is 800 mm

### Study on surrounding rock support of auxiliary withdrawal channel

#### The deviatoric stress distribution of surrounding rock in coal pillar recovery working face

**Fig. 25:**
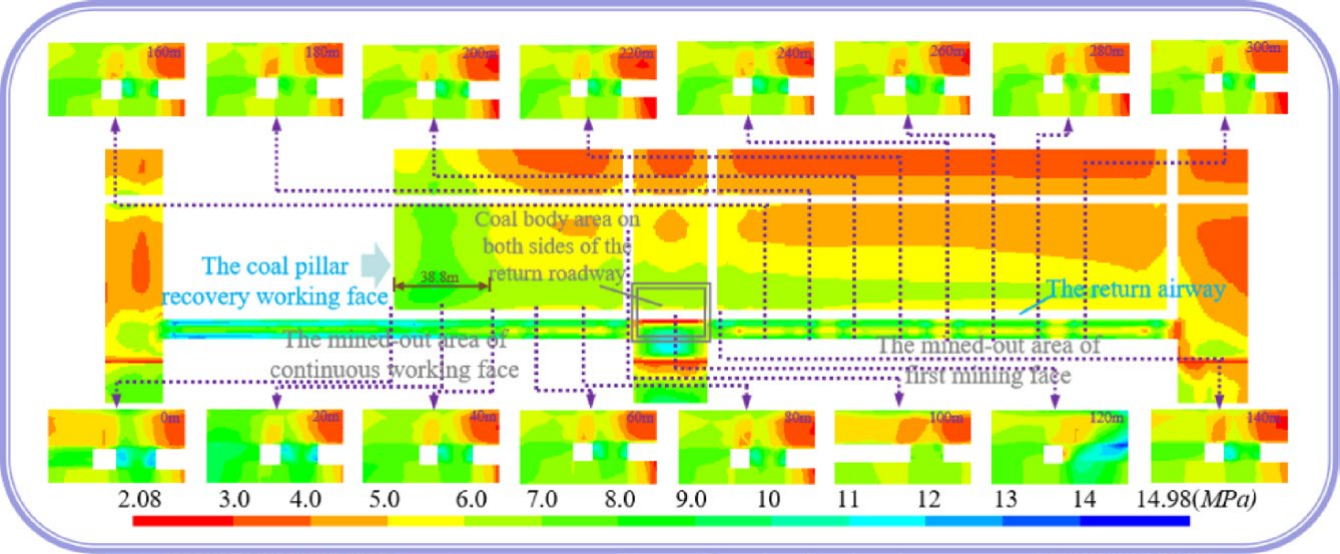
The deviatoric stress cloud diagram of surrounding rock of return airway.

The auxiliary withdrawal channel is not only affected by the end mining stage of the coal mining working face, but also needs to be retained as the return airway of the coal pillar recovery working face. The return airway (the original auxiliary withdrawal channel) of the coal pillar recovery working face is also affected by the mining of the coal pillar recovery working face. Therefore, it is necessary to study the deviatoric stress distribution of the surrounding rock of the return airway during the mining process of the coal pillar recovery working face. The distribution cloud diagram of the deviatoric stress of the surrounding rock in the axial direction of the return airway of the coal pillar recovery working face is shown in Fig. 25.

**Fig. 26:**
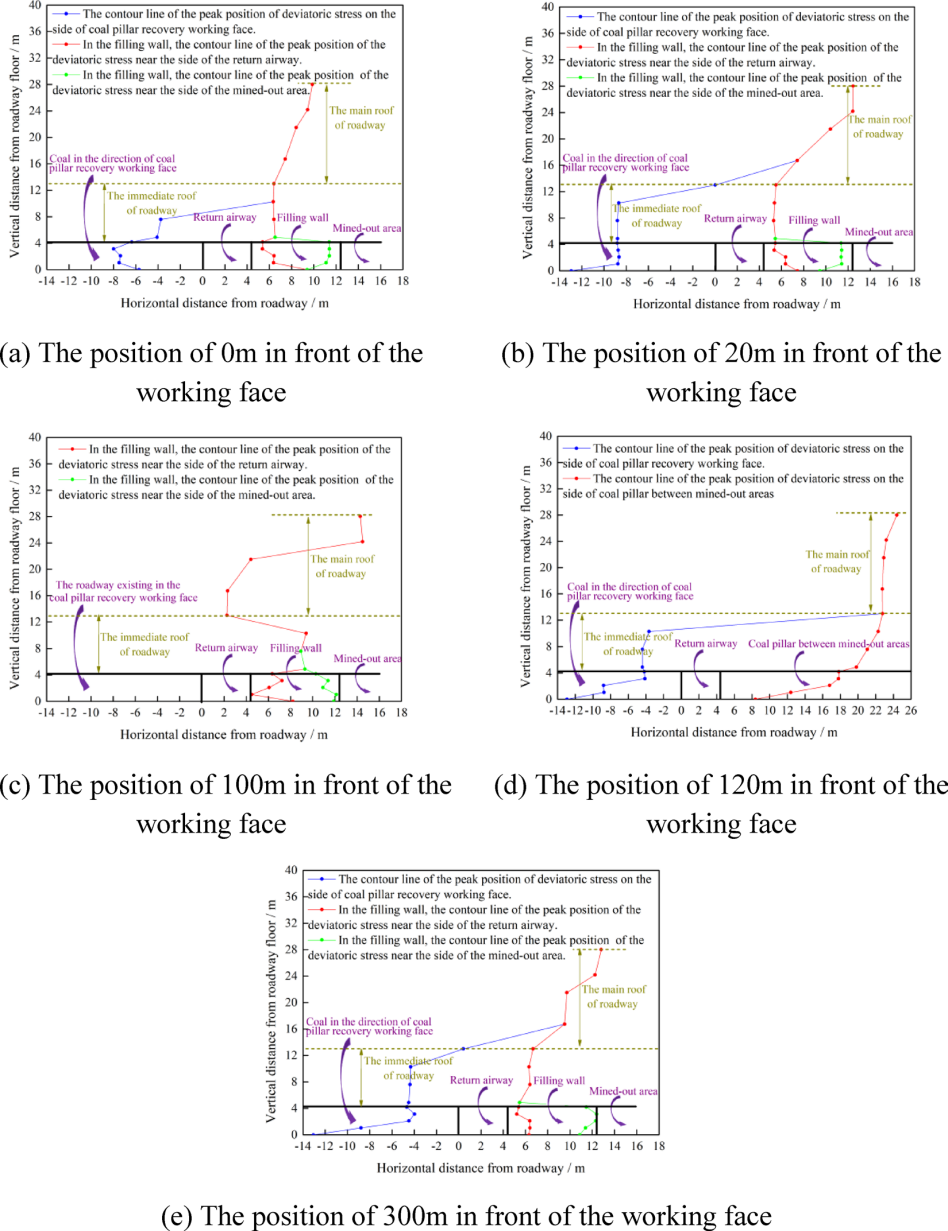
The contour lines of the peak position of the deviatoric stress at different positions in front of coal pillar recovery working face.

As shown in Fig. 25, the influence range of the advance deviatoric stress of the coal pillar recovery working face is about 38.8 m. In the area where one side of the return airway is the filling wall and the other side is the coal body, the deviatoric stress is mainly borne by the filling wall. In the area where both sides of the return airway are coal bodies, the deviatoric stress is mainly borne by the coal pillar between the mined-out areas, and the phenomenon of deviatoric stress concentration occurs. Because the deviatoric stress distribution of the surrounding rock of the return airway is changed by the mining influence of the coal pillar recovery working face. In order to study and design the support parameters of the surrounding rock of the return airway, five sections with the distance of 0 m 20 m, 100 m, 120 m and 300 m from the coal pillar recovery working face were selected to draw the peak position of the deviatoric stress in the surrounding rock of the return airway of these five sections, as shown in Fig. 26.

As shown in Fig. 26, due to the different cross-section positions of the return airway, the surrounding rock of the roadway can be divided into three forms. 1.One side is the coal pillar recovery working face, and the other side is the filling wall. 2.One side is the coal pillar recovery working face, and the other side is the coal pillar between mined-out areas. 3.One side is the roadway existing in the coal pillar recovery working face, and the other side is the filling wall. At the same time, the return airway is affected by mining of coal pillar recovery working face, and the contour line distribution of the deviatoric stress peak of the surrounding rock of the return airway is more complicated. With the decrease of the distance from the coal pillar recovery working face, the peak contour line of the deviatoric stress on the side of the coal pillar recovery working face moves to the direction of the return airway, the peak contour line of the deviatoric stress in the filling wall moves to the inside of the wall, and the peak contour line of the deviatoric stress above the roadway moves to the direction of the return airdway. Therefore, the surrounding rock of the auxiliary withdrawal channel is affected by two times of mining. The mining influence of the coal pillar recovery working face further aggravates the damage of the surrounding rock of the return airway.

#### Supporting parameters of surrounding rock

**Fig. 27:**
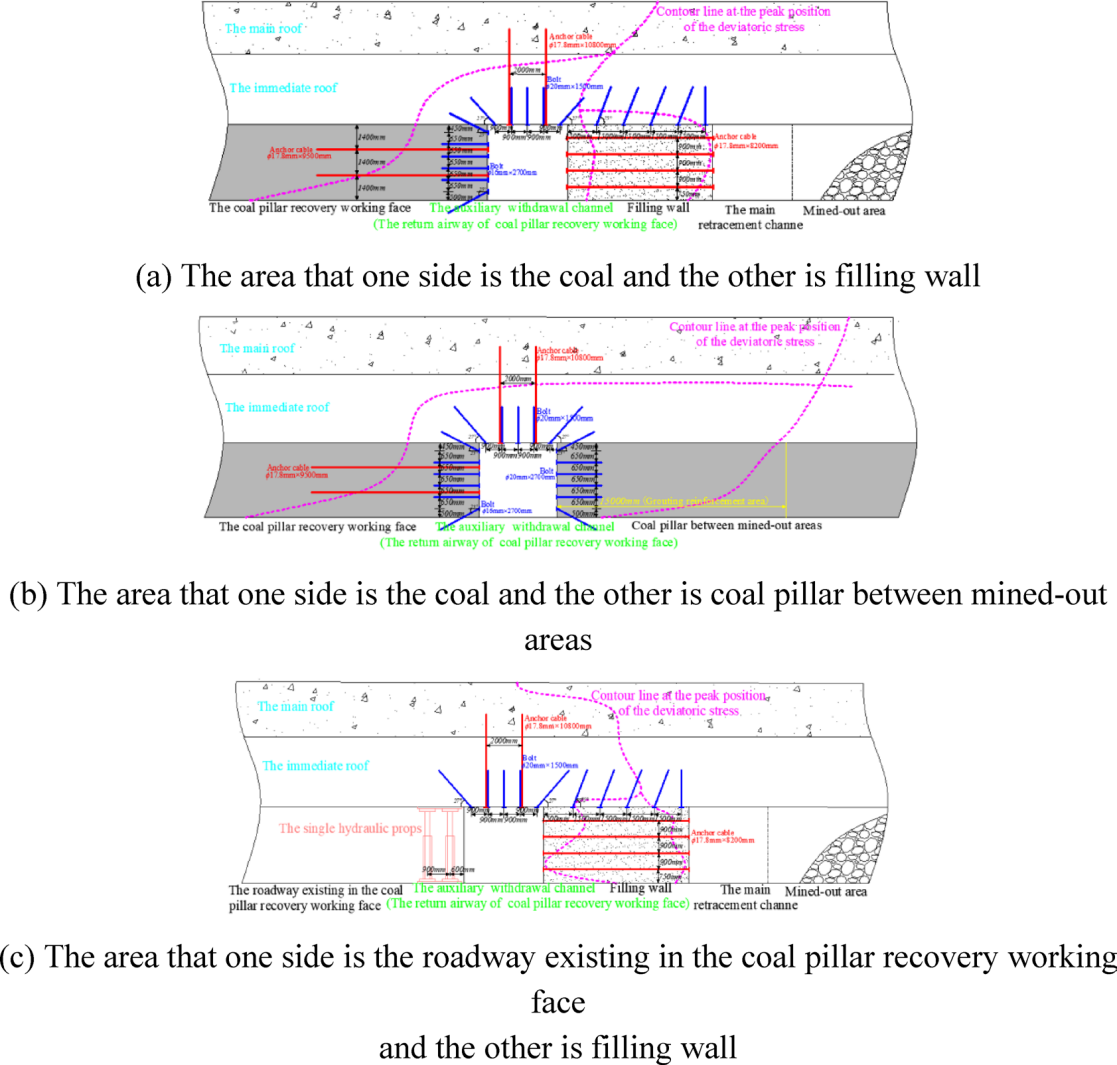
Section diagram of supporting structure of surrounding rock in return airway.

**Fig. 28:**
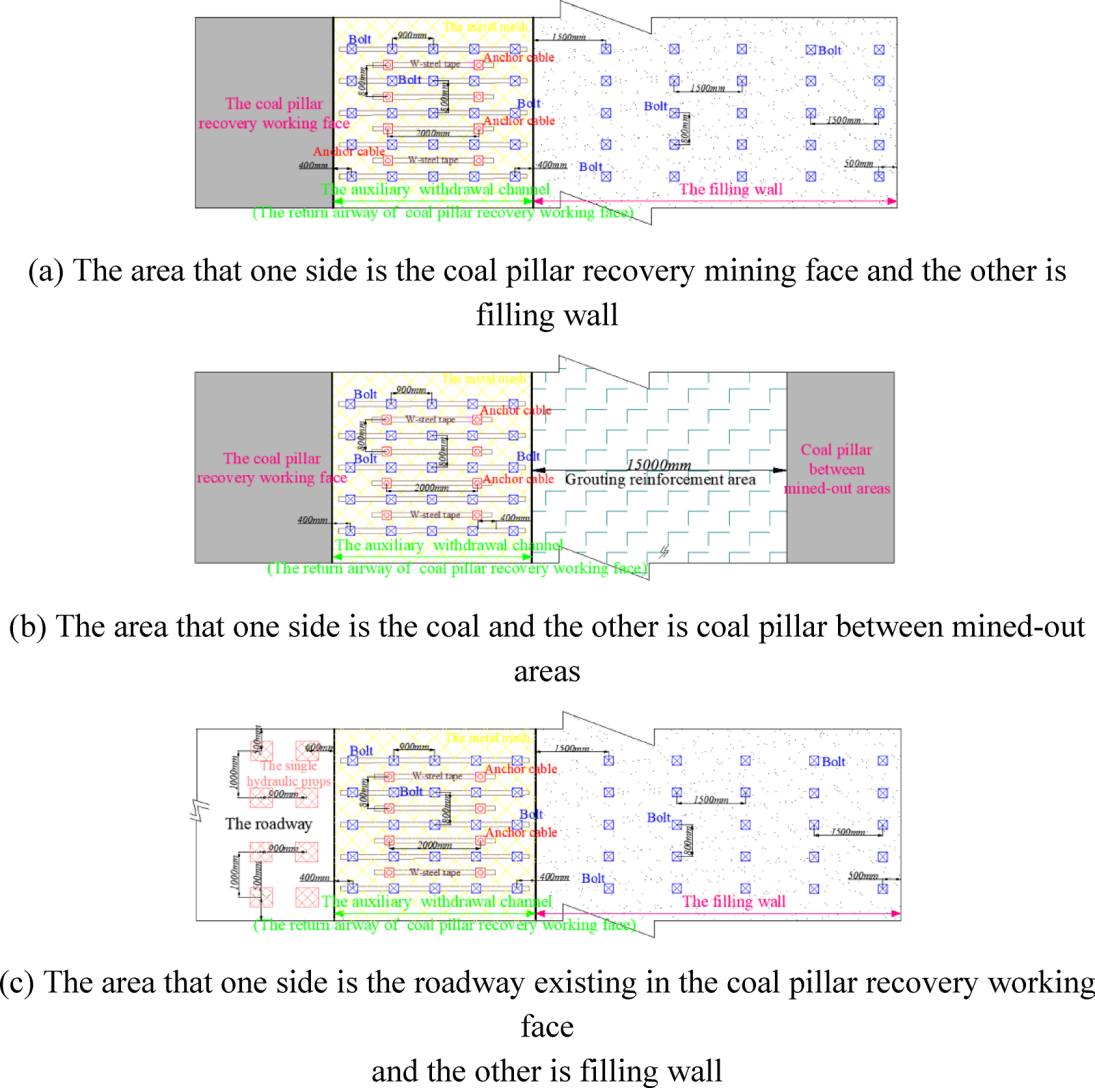
Planar graph of supporting structure of surrounding rock in return airway.

Because the auxiliary withdrawal channel is a part of the return airway of the coal pillar recovery working face, this summary uniformly calls the auxiliary withdrawal channel as the return airway. Combined with the distribution law of the contour line at the peak position of the deviatoric stress, the surrounding rock of the return airway adopts different supporting structures in different regions. The sectional diagram and planar graph of the supporting structure are shown in Figs. 27 and 28.

The area that one side is the coal pillar recovery working face and the other side is filling wall, which supporting parameters in this region are as follows:

1. Supporting parameters of roadway roof
  The five *φ*20mm rebar bolts are designed, and the length of the rebar bolt is *1500 mm*. The spacing between the bolts is 900 mm. The row spacing between the bolts is 800 mm. Two *φ*17.8 mm high pre-tightening force steel cable anchor cables are designed, and the length of the anchor cable is 10800 mm. The spacing between the anchor cables is 2000 mm, and the row spacing between the anchor cables is 800 mm. The interval arrangement between anchor cable and anchor bolt is adopted.
2. Supporting parameters of side of the coal pillar recovery working face

The six *φ*16mm reinforced fiber glass bolts are designed, and the length of the reinforced fiber glass bolt is 27*00 mm*. The spacing between the bolts is *650 mm*, and the row spacing between the bolts is 800 mm. Tow *φ*17.8 mm steel cable anchor cables are designed. The length of the anchor cable is 9500 mm, the spacing between the anchor cables is 1400 mm, and the row spacing between the anchor cables is 800 mm.

The area that one side is the coal pillar recovery working face and the other side is coal pillar between mined-out areas, which supporting parameters in this region are as follows:

1. Supporting parameters of roadway roof

The five *φ*20mm rebar bolts are designed, and the length of the rebar bolt is *1500 mm*. The spacing between the bolts is 900 mm, and the row spacing between the bolts is 800 mm. Two *φ*17.8 mm high pre-tightening force steel cable anchor cables are designed. The length of the anchor cable is 10800 mm, the spacing between the anchor cables is 2000 mm, and the row spacing between the anchor cables is 800 mm. The interval arrangement between anchor cable and anchor bolt is adopted.

2. Supporting parameters of the side of the coal pillar recovery working face

The six *φ*16mm reinforced fiber glass bolts are designed, and the length of the reinforced fiber glass bolt is 2700 mm. The spacing between the bolts is 650 mm, and the row spacing between the bolts is 800 mm. Tow *φ*17.8 mm steel cable anchor cables are designed. The length of the anchor cable is 9500 mm, the spacing between the anchor cables is 1400 mm, and the row spacing between the anchor cables is 800 mm.

3. Supporting parameters of the side of the coal pillar between mined-out areas

The six *φ*16mm reinforced fiber glass bolts are designed, and the length of the reinforced fiber glass bolt is 2700 mm. The spacing between the bolts is 650 mm, and the row spacing between the bolts is 800 mm. The depth of grouting into the coal pillar between mined-out areas is 15000 mm.

The area that one side is the roadway existing in the coal pillar recovery working face and the other is filling wall, which supporting parameters in this region are as follows:

1. Supporting parameters of roadway roof

The five *φ*20mm rebar bolts are designed, and the length of the rebar bolt is *1500 mm*. The spacing between the bolts is 900 mm, and the row spacing between the bolts is 800 mm. Two *φ*17.8 mm high pre-tightening force steel cable anchor cables are designed. The length of the anchor cable is 10800 mm, the spacing between the anchor cables is 2000 mm, and the row spacing between the anchor cables is 800 mm. The interval arrangement between anchor cable and anchor bolt is adopted.

2. Supporting parameters of direction of existed roadway in the coal pillar recovery working face

The double-row single hydraulic props are arranged in the roadway, with a spacing of 1100 mm and a row spacing of 900 mm.

## Engineering application

**Fig. 29:**
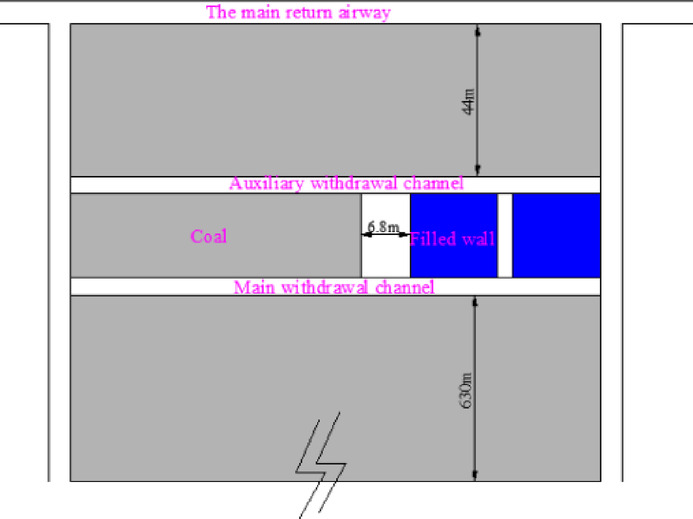
The construction flow chart.

When the 3302 working face is 630 m away from the stopping position, the main withdrawal channel and the auxiliary withdrawal channel are excavated. The surrounding rock of the withdrawal channel is supported.The distance between the auxiliary withdrawal channel and the main roadway is 44 m. The distance between the main withdrawal channel and the auxiliary withdrawal channel is 8 m. After the excavation of the main withdrawal channel and the auxiliary withdrawal channel is completed, the coal pillar between the main withdrawal channel and the auxiliary withdrawal channel is mined. The wall is filled at 6.8 m behind the mined coal pillar. The mining speed of the coal pillar is 2 m per day, and the filling speed of the wall is 2 m per day. The flow chart is shown in Fig. 29. The filling material used is composed of cement, sand, rubble, water, concrete pumping agent (FDN-3), early strength water reducing agent (EHD) and sodium meta aluminate (P0102). Raw material ratio and water cement ratio are shown in Table 4.

**Table. 4:** The proportioning of filling materials.

Material	cement	sand	rubble	water	FDN-3	EHD	P0102
Mass(kg)	520	800	850	226	1.2	4	0.3
Proportion of mass(%)	20.86	34.1	35.39	9.41	0.05	0.2	0.01

**Fig. 30:**
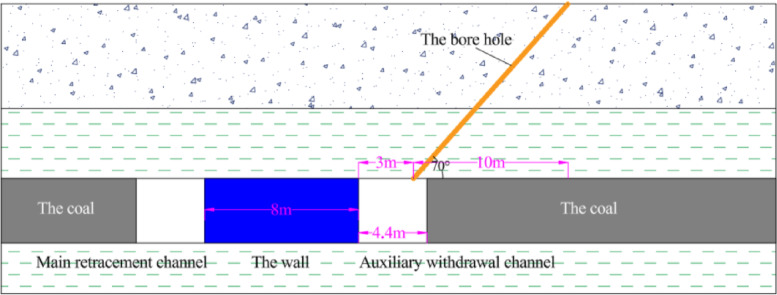
Layout diagram of pre-cut roof.

The coal pillar between the main withdrawal channel and the auxiliary withdrawal channel was replaced by the wall while supporting the wall. When the coal pillar was completely replaced, hydraulic fracturing was performed on the roof of the main withdrawal channel. The diameter of the borehole was 120 mm, the angle of the borehole was 70^°^, and the distance between the boreholes was 3 m, as shown in Fig. 30.

**Fig. 31:**
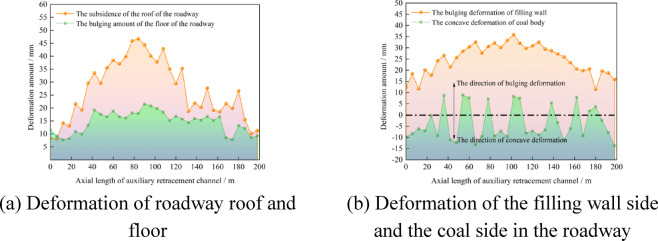
Surrounding rock deformation of the retained auxiliary withdrawal channel.

On the 126th day after the mining of the 3302 working face was completed, the deformation of the surrounding rock of the retained auxiliary mining roadway was measured, as shown in Fig. 31.

As shown in Fig. 31, in the retained auxiliary withdrawal channel, the subsidence of the roof was between 8.1 mm ~ 46.7 mm, the bulge of the floor was between 7.7 mm ~ 21.5 mm, and the bulge of the filling wall was between 12.3 mm ~ 35.9 mm. The solid coal was a concave and convex irregular deformation, and the depression of the solid coal was between 0.3 mm ~ 13.7 mm, and the bulge of the solid coal was between 1.8 mm ~ 8.9 mm. The surrounding rock deformation of the roadway remains stable and met the requirements of subsequent use. And the retained retracement channel is used as a space for storing materials.

**Fig. 32:**
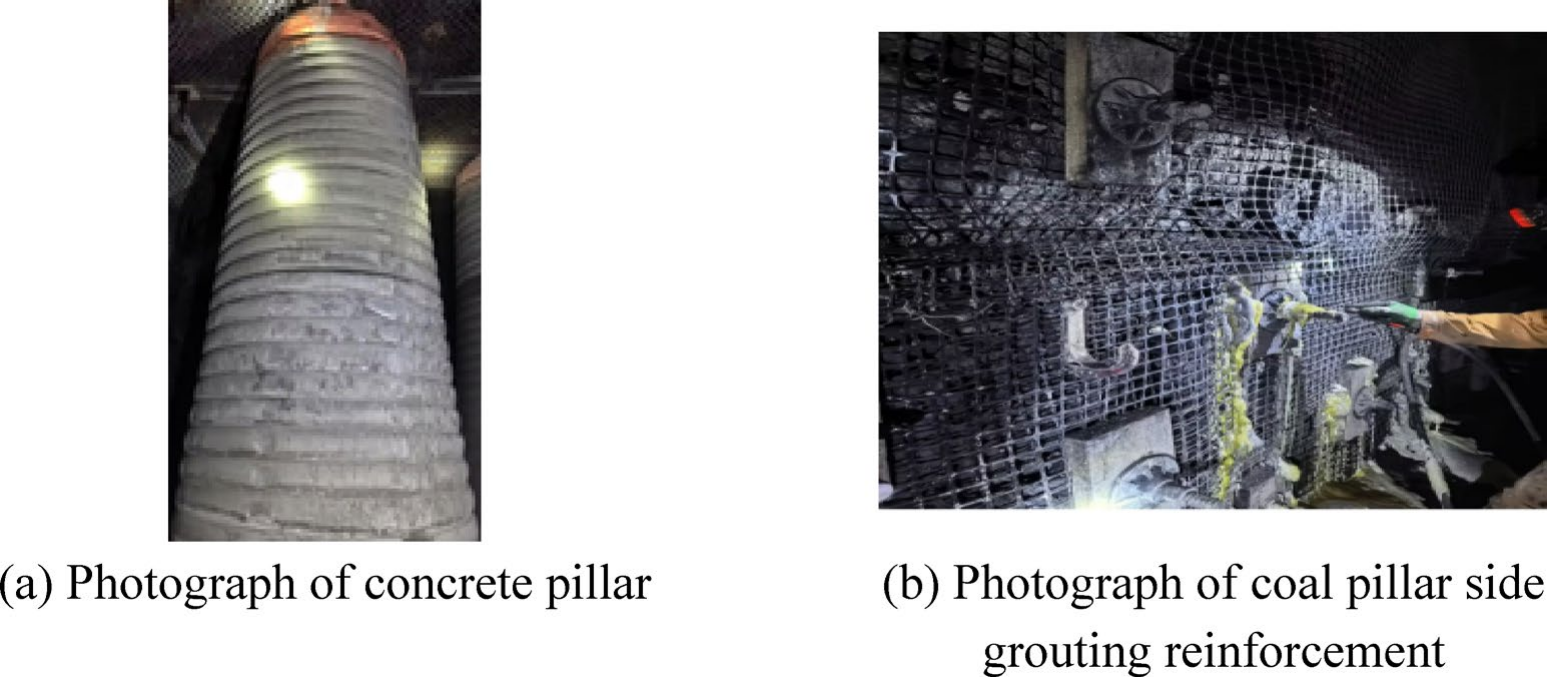
Supporting structure of roadway of area that one side is the coal pillar recovery working face and the other side is coal pillar between mined-out areas.

The same method was used to mine the 3303 mining coal working face. After the coal mining equipment of the 3303 mining coal working face was migrated, a roadway was excavated to connect the auxiliary withdrawal channel of the 3303 mining coal working face and the auxiliary withdrawal channel of the 3302 mining coal working face. In order to prevent the destruction of the surrounding rock of this section of the roadway, the concrete pillars were arranged in the roadway, and the concrete pillars were arranged in two rows. The row spacing and spacing of the concrete pillars were *2.4 m* and *1.4 m*, respectively, as shown in Fig. 32. Therefore, the surrounding rock support system of the withdrawal channel is the combined control technology of ‘ pre-cutting roof + high water material pillar + grouting reinforcement + single pillar + metal mesh + steel ladder belt + bolt + anchor cable ‘ in different areas.

**Fig. 33:**
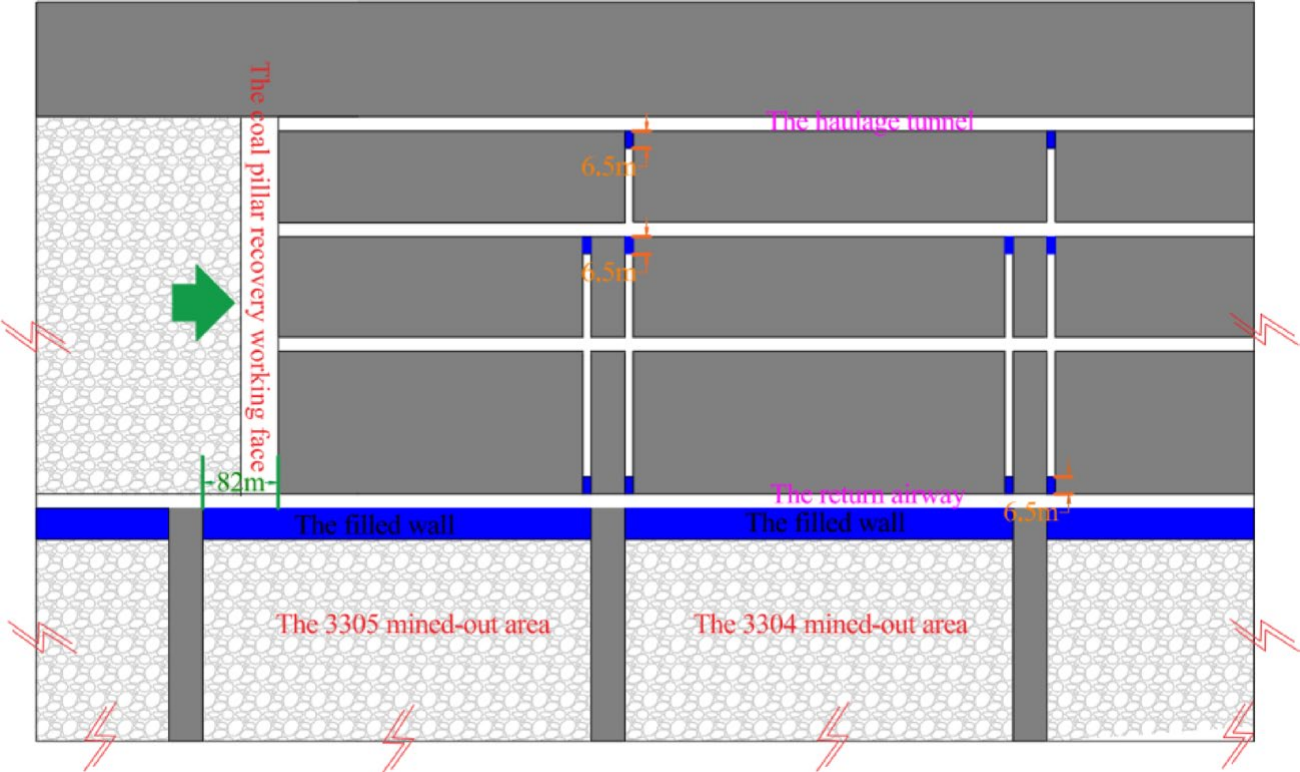
The advancing position of coal pillar recovery working face.

**Fig. 34:**
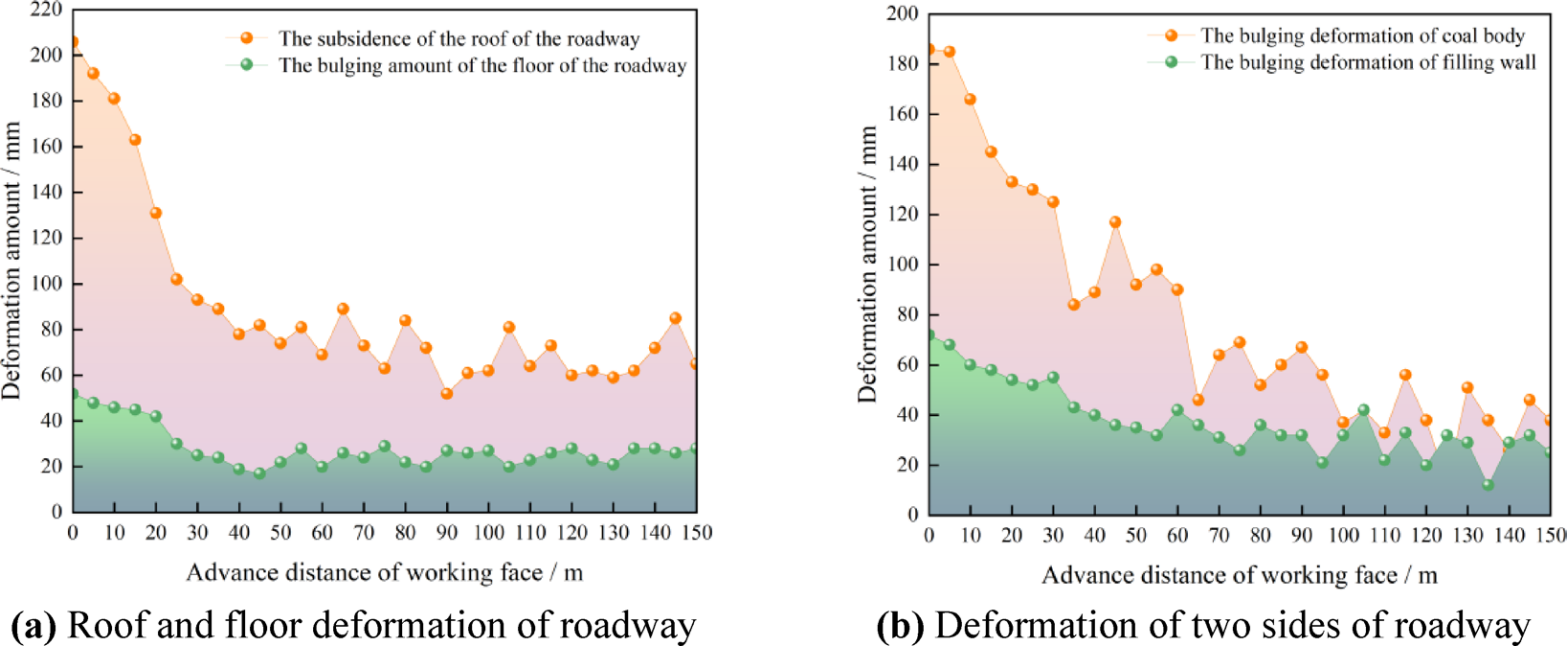
Surrounding rock deformation of roadway.

After all the coal mining faces were mined, the remained coal pillars were recovered. Before recovering the coal pillars, the concrete walls were used to block the roadway left in the coal pillar to prevent the airflow disorder. When the coal pillar recovery working face was advanced to the position of 3305 goaf (Fig. 33a), the surrounding rock deformation of the return air roadway in the range of 150 m in front of the working face, that was, the surrounding rock deformation of the retained auxiliary retracement, was shown in Fig. 34.

As shown in Fig. 34, the mining influence range of the coal pillar recovery working face was about 38 m, and the maximum deformation of the roof and floor of the roadway was 206.2 mm and 52.1 mm, respectively. The maximum deformation of the coal side and the wall side of the roadway was 186.1 mm and 72.4 mm, respectively. The deformation of roadway surrounding rock meets the needs of safe production. The pre-excavated double withdrawal channel technology without coal pillar abandonment breaks through the traditional mode and realizes the integrated process of ‘ retracement channel excavation-equipment transfer-reservation of retracement channel-auxiliary production of retracement channel-retracement channel recovery of coal pillar ‘. In addition, using this technology, the amount of recoverable coal resources in 3# coal seam would increase by 747000t. The economic benefits will increase by 117 million yuan.

## Conclusion

1. Using the stress superposition principle of two adjacent unequal diameter circular holes, the limit size of the coal pillar between the coal mining face and the main retracement channel is calculated. The mechanical model of the roof is established, and the reasonable size of the coal pillar between the main and auxiliary withdrawal channels is analyzed.
2. Using the deviatoric stress distribution law of surrounding rock, the upper and lower limits of the surrounding rock support range of the retracement channel are constructed, and different support structures are used in different areas of the retracement channel.
3. The pre-excavated double withdrawal channel technology without coal pillar abandonment breaks through the traditional mode and realizes the integrated process of ‘ retracement channel excavation-equipment transfer-reservation of retracement channel-auxiliary production of retracement channel-retracement channel recovery of coal pillar ‘.
4. Using pre-excavated double withdrawal channel technology without coal pillar abandonment, the amount of recoverable coal resources in 3# coal seam will increase by 747000t. The economic benefits will increase by 117 million yuan.

## Data Availability

All data generated or analysed during this study are included in this published article.
